# Moving from Classical Ru-NHC to Neutral or Charged Rh-NHC Based Catalysts in Olefin Metathesis

**DOI:** 10.3390/molecules21020177

**Published:** 2016-01-30

**Authors:** Albert Poater

**Affiliations:** Institut de Química Computacional i Catàlisi and Departament de Química, Universitat de Girona, Campus Montilivi, 17071 Girona, Catalonia, Spain; albert.poater@udg.edu; Tel.: +34-972-418-358

**Keywords:** olefin metathesis, ruthenium, rhodium, *N*-heterocyclic carbene, interchange, initiation, DFT

## Abstract

Considering the versatility of oxidation states of rhodium together with the successful background of ruthenium-*N*-heterocyclic carbene based catalysts in olefin metathesis, it is envisaged the exchange of the ruthenium of the latter catalysts by rhodium, bearing an open-shell neutral rhodium center, or a +1 charged one. In the framework of *in silico* experiments, density functional theory (DFT) calculations have been used to plot the first catalytic cycle that as a first step includes the release of the phosphine. DFT is, in this case, the tool that allows the discovery of the less endergonic reaction profile from the precatalytic species for the neutral catalyst with respect to the corresponding ruthenium one; increasing the endergonic character when dealing with the charged system.

## 1. Introduction

During the last three decades, thousands of papers have presented and described the olefin metathesis catalysis, by experimental synthesis and characterization [1], as well as in the validation of computational protocols [2,3,4,5,6,7,8,9]. However, neither a general catalyst for any metathesis reaction [10,11,12] nor perfect rules are available to predict the behavior of a given catalyst has been achieved [13,14], bearing the efforts in characterizing the decomposition reactions [15]. However, olefin metathesis has successfully achieved the goal of organic synthesis that consists of reactions that drive to the formation of carbon-carbon bonds [16,17,18], and provides a route to unsaturated molecules. Basically, the area of ruthenium-catalyzed [19,20] olefin metathesis reactions centers the last industrial applications during the last decade [21], inspired by the previous discovery first by Grubbs *et al.* of well-defined Ru-based catalysts, such as (PCy_3_)_2_Cl_2_Ru=CHPh [22], together with the substitution of one phosphine group by a N-heterocyclic carbene, NHC [23,24], increasing strongly the activity [25,26]. Once a better understanding of the performance of such catalysts was achieved, a rational design of new more active catalysts was envisaged [27,28] Despite experimental [29,30,31] and theoretical [32,33] insights during the last two decades, demonstrating the mechanism bears a metallacycle as suggested by Chauvin [34], still there are chances to improve the catalysis in olefin metathesis [35,36], mainly due to the undesired parallel reactions [37,38,39], or low capability to deal with water or alcohols as solvents [40,41].

Even though molybdenum [42,43] and basically ruthenium are the metals reference in olefin metathesis there are several good results bearing tungsten, with several other attempts including iron [44,45], osmium [46], or rhodium [47,48,49,50]. However, none has overcome the performance of Mo and Ru-based catalysts. To this end, computational techniques are a popular tool to screen novel catalyst architectures more rapidly and to explore their full potential as efficient catalysts. In the past, several promising new compounds were proposed by density functional theory (DFT) calculations [51,52]. In the present study, DFT calculations are used again to investigate the activation mechanism of *N*-heterocyclic carbene (NHC)-Rh based catalysts to understand the effect of replacing Ru by Rh [53]. To sum up, this study gives insight and at least opens a door towards a proposal of a new family of olefin metathesis catalysts [54], bearing rhodium as the metal catalyst.

## 2. Results

**Scheme 1:**
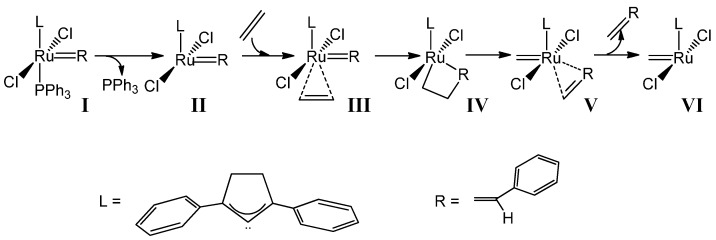
Mechanism of the olefin metathesis for Ru-NHC based complexes.

Bearing the classical Ru(SIMes)Cl_2_(=CHPh)PPh_3_ olefin metathesis catalyst, by density functional theory (DFT) calculations, it was tested the effect of replacing Ru by Rh, affording either the neutral open-shell duplet or the +1 charged closed shell Rh(SIMes)Cl_2_(=CHPh)PPh_3_ system. To evaluate the free energy surface of Rh-NHC based catalysts, it was explored the mechanism displayed in Scheme 1, which basically initially consists of the release of the phosphine group, with the consequent generation of a 14-electron species **II**, which binds to an olefin, coordinated cis to the alkylidene [55,56]. The exchange of the leaving group by an olefin is found to be mainly dissociative [57,58], but with some alternative associative and concerted mechanisms [59,60]. The next metallacycle intermediate **IV** is due to the reaction of the olefin with the alkylidene moiety. The next steps after the metallacycle are identical by quasi-symmetry with respect to the previous ones.

**Figure 1:**
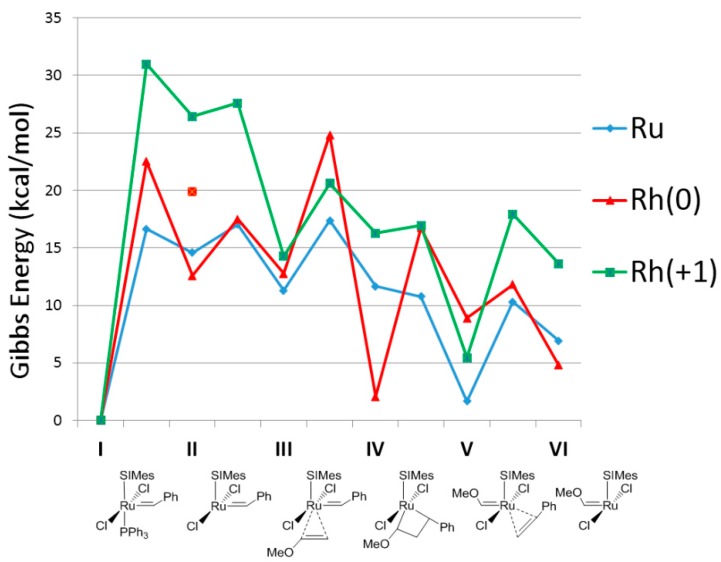
Computed stationary points for the olefin metathesis reaction pathway for M(SiMes)Cl_2_(=CHPh)PPh_3_ with methoxyethene (M = Ru in blue, Rh(0) in red, Rh(+1) in green; energies in kcal/mol, selected distances in Å, the imaginary frequencies characterizing the transition states structures are given in brackets; for Rh(0) the transition state **I**–**III** is also included in red).

Figure 1 includes the free energy surface for Rh(SIMes)Cl_2_(=CHPh)PPh_3_ (both neutral and +1 charged) catalyzed metathesis with vinyl ethers, specifically the energy profile for the first turnover of this reaction. Although the latter substrate is known to lead to catalytically inactive Fischer-type carbenes after a single turnover it provides a straightforward reaction with which to study the initiation kinetics, either experimentally [61,62] or theoretically [54]. However, although the kind of olefin may not affect the first turnover, it might affect the propagation steps [42,43,54]. In Figure 1 the energy values are compared to the corresponding Ru(SIMes)Cl_2_(=CHPh)PPh_3_ analogue. Focusing on the first turnover, using the neutral [Rh(SIMes)Cl_2_(=CHPh)PPh_3_] as a catalyst, Figure 1 gives the result that the simplest dissociative pathway starts with the initial loss of PPh_3_ ligand in precatalyst **I**, forming the catalytically active 14e species **II**, which is placed 12.6 kcal/mol above **I**, requiring the overcoming of a barrier of 22.5 kcal/mol. Bearing a low barrier of 4.9 kcal/mol, the relative low stability of species **II** assists the next olefin coordination to the metal center to give the intermediate **III**, which is practically isoenergetic with respect to the 14e species **II**. However, the concerted initiation step that links **I** directly to **III** here turns out to be favored, defining an energy barrier 2.6 kcal/mol lower than the upper barrier of the dissociative mechanism that corresponds to the transition state **I**-**II**.

Once the labile ligand is released and as the entering olefin is bonded to the metal, the still relatively unstable intermediate **III** is prone to collapse to the much more stable metallacycle intermediate **IV**, lying 10.8 kcal/mol below **III**. However, the barrier of 12.0 kcal/mol might be a bottleneck of the first turnover, because such step bears its upper energy point. In contrast, the upper energy barrier corresponds to the following ring opening of metallacycle **IV**, which results in the formation of another coordination intermediate **V** with a cost of 14.7 kcal/mol from **IV**. From an energetic point of view, intermediate **V** is 3.9 kcal/mol more stable relative to **III**, and the next release of the alkene might be rather facile because it requires just 2.9 kcal/mol, finally leading to the formation of second 14e species **VI**, which is interestingly 11.8 kcal/mol lower in energy with respect to the first 14e species **II**, suggesting that the catalytically active pathway is exothermic.

The energy profile of the first olefin metathesis reaction turnover for [Rh(SIMes)Cl_2_(=CHPh)PPh_3_]^+1^. The release of the labile phosphine ligand to form a 14-electron species (**II**) is rather expensive, endergonic by 26.4 kcal/mol, apart from the barrier placed even 4.6 kcal/mol higher in energy. For the charged Rh-based catalyst the concerted step **I**-**III** is even 2.9 kcal/mol less stable. Next, the remaining steps for coordination of the olefin (**III**), formation of the metallacycle (**IV**), and next coordination intermediate (**V**), the release of the benzylidene moiety and finally the formation of the 14e carbene (**VI**) follow a decay of energy. Notably, to open the metallacycle **IV** costs only 0.6 kcal/mol, whereas to go back costs 4.3 kcal/mol. Further, the release of the benzylidene moiety (**V**) and finally the formation of the 14e carbene (**VI**) are slightly complicated since this second 14e species is formed, overcoming a barrier of 12.5 kcal/mol together with an endergonic loss of 8.2 kcal/mol.

## 3. Discussion

**Figure 2:**
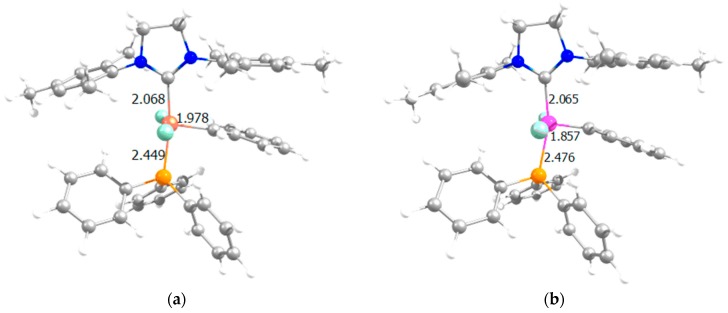
Species **I** for: (**a**) Rh and (**b**) Ru (selected distances in Å).

**Figure 3:**
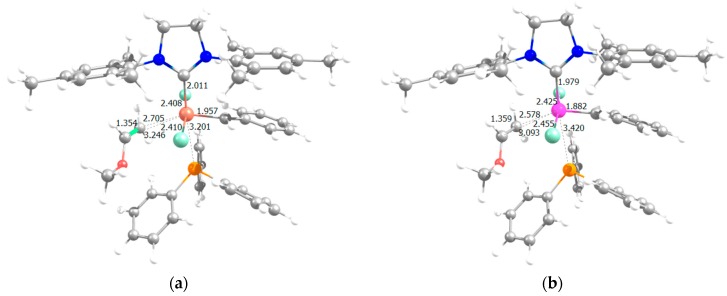
Transition state **I**-**III** for: (**a**) Rh and (**b**) Ru (selected distances in Å, the imaginary frequencies characterizing the transition states structures are 36.5i and 32.9i, respectively).

Figure 2 displays species **I** for neutral Rh- and Ru-based catalysts, and Figure 3 includes the sterically crowded transition state **I**-**III** together with the corresponding stationary point bearing ruthenium, pointing out that for Ru, experimentally, this concerted mechanism is not feasible, but is dissociative [54]. For, Ru the interchange mechanism became favored when increasing the sterical hindrance of the ylidene ligand, for instance, phenylidene by indenylidene moiety. To unravel the reason for the preference for the interchange mechanism the geometrical analysis of species **I** does not show any difference. Take for instance the calculated percent buried volume (%V_Bur_) [63] around the metal due to the NHC ligand being exactly the same bearing any of both metals (30.8). However, the specific analysis of the quadrants revealed a slight difference: for both metals, three out of four quadrants are generously occupied with a less occupied quadrant for Rh (%V_Bur_ = 26.3, 27.4 for Rh and Ru, respectively), which might help the next insertion of the entering olefin, despite the difference not being significantly different (see also Table S2 and Figure S1 for further details). However, the longer Rh-C_phenylidene_ bond (1.978 and 1857 Å for Rh and Ru, respectively) allocates the right environment around the metal for the exchange of the phosphine by the entering olefin at the same time (see Table S1 for further geometrical details of species **I** bearing Rh or Ru). Going further into structural details, this hypothesis is confirmed by a Mayer Bond Order (MBO) analysis [64] of the Rh- and Ru-based precatalyst **I**. MBO values reveal a much weaker metal–C_phenylidene_ bond for Rh (1.058 for Rh *vs.* 1.792 for Ru), together with a weaker SIMes-metal bond (0.782 for Rh *vs.* 0.925 for Ru) as well, bearing a similarly strong M–P bond (0.713 for Rh *vs.* 0.701 for Ru). Thus, the main structural difference is that the metal–C_phenylidene_ bond is much weaker for Rh, with a MBO that defines a simple bond instead of the double bond that bears the Ru-based precatalyst **I**. Thus this difference is translated into a larger flexibility around the rhodium, facilitating the concerted transition state **I**-**III**. This rationalizes the preference for the interchange mechanism rather than the dissociative one for Rh. 

Mechanistically, the +1 charged rhodium catalyst also displays the same relative advantages with respect to the neutral catalyst, although the neutral is favored due to its exergonicity and the less difficult phosphine dissociation. It is worth mentioning that all complexes in the above studied Rh catalyzed reaction pathways exhibited singlet ground state, as ruthenium homologous mechanism [50,65,66,67,68], but differently with respect to iron [42,43]. However, here the rhodium center might be disproportionate, or lose one chloride, like Castarlenas *et al.* have recently demonstrated [53]. For the sake of clarity, the comparison between the neutral Ru and Rh based catalysts in Figure 1 reveals a promising exergonicity of 2.1 kcal/mol for the latter species. Further, the first olefin metathesis reaction turnover showed acceptable energetic stability of all involved intermediates together with reasonable low energy barriers, suggesting that the Rh calculated profile might afford a potentially active catalyst.

## 4. Materials and Methods

All the DFT static calculations were performed with the Gaussian09 set of programs [69]. For geometry optimization, the well-established and computationally fast GGA functional BP86 was used [70,71]. Geometry optimizations were performed without symmetry constraints, while the located stationary points were characterized as minima or transition state by analytical frequency calculations. The electronic configuration of the molecular systems was described with the standard split-valence basis set with a polarization function of Ahlrichs and co-workers for H, C, O, P, and Cl (SVP keyword in Gaussian) [72]. For Ru, we used the small-core, quasi-relativistic Stuttgart/Dresden effective core potential, with an associated valence basis set contracted (standard SDD keywords in Gaussian 09) [73,74,75]. Zero point energies and thermal corrections calculated at the BP86 level were added to the M06 in solvent energies [76] to approximate free energies in solvent using the triple-ζ valence plus polarization basis set for main group atoms (TZVP keyword in Gaussian). Since entropic contribution calculated within the ideal gas approximation at P = 1 atm is likely exaggerating the expected values for the dissociative steps in the condensed phase [77,78,79,80,81,82,83,84], all the thermochemical analyses were performed at P = 1354 atm and T = 298.15 K, as suggested by Martin *et al.* [85,86]. Solvent effects were included with the polarizable continuous solvation model PCM using dichloromethane as solvent [87,88]. The M06 energy calculations were carried out with the scf=tight, and integral(grid=ultrafinegrid) keywords. This approach was recently shown to be particularly effective in the modelling of Ru-promoted olefin metathesis [89], however might not be a reference method for charged rhodium species.

%VBur Calculations: The buried volume calculations were performed with the SambVca package developed by Cavallo *et al.* [61]. The radius of the sphere around the metal center was set to 3.5 Å, while for the atoms it was adopted the Bondi radii scaled by 1.17, and a mesh of 0.1 Å was used to scan the sphere for buried voxels. The steric maps were evaluated with a development version of the SambVca package [90].

## 5. Conclusions

To sum up, the first turnover of olefin metathesis, using a homogenous, theoretically predicted neutral Rh-based catalyst with methoxyethene was described by means of DFT calculations. The reasonable energy barriers along the reaction pathway, together with the slightly higher exothermicity for Rh makes Rh a potential metal substitute for Ru despite being a precious metal. However, the upper energy point is 8.2 kcal/mol higher in energy with respect to Ru, and does not correspond to the phosphine release, but to the closure of the metallacycle. Moreover, the Rh-based catalyst is appealing to get a new family of catalysts that clearly bear an interchange mechanism for the direct transformation of the precatayst to the coordination intermediate, without any need to increase the size of the ylidene ligand, from phenylidene to indenylidene.

## Supplementary Materials

The following are available online at www.mdpi.com/1420-3049/21/2/177, Table S1: Cartesian coordinates, 3D view, and energies of all the species discussed in this work; Table S2 and Figure S1: Data for %V_Bur_ analysis.

## Abbreviations

The following abbreviations are used in this manuscript:

SIMes: 1,3-Bis(2,4,6-trimethylphenyl)-4,5-dihydroimidazol-2-ylidene
