# Supplementary material for: Moving from Classical Ru-NHC to Neutral or Charged Rh-NHC Based Catalysts in Olefin Metathesis
Source: Molecules. 2016 Jan 30;21(2):177. doi: 10.3390/molecules21020177 (PMC6273139; doi:10.3390/molecules21020177)
Supplement: Supplementary file 1 [file molecules-21-00177-s001.docx]

Supplementary Materials: Moving from Classical Ru-NHC to Neutral or Charged Rh-NHC Based Catalysts in Olefin Metathesis

Albert Poater

**Table S1.** Coordinate data sets and absolute energy (in a.u.), 3D structure for DFT optimized complexes with selected distances (in Å).

| **Rh-Id** | |
| --- | --- |
| 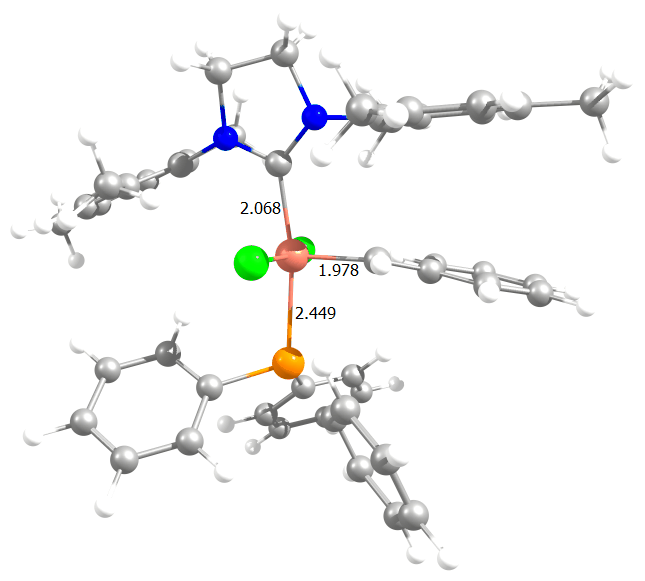  Zero-point correction= 0.779957 (Hartree/Particle)  Thermal correction to Energy= 0.834865  Thermal correction to Enthalpy= 0.835809  Thermal correction to Gibbs Free Energy= 0.685232  Sum of electronic and Zero-point Energies= −3263.132801  Sum of electronic and thermal Energies= −3263.077893  Sum of electronic and thermal Enthalpies= −3263.076949  Sum of electronic and thermal Free Energies= −3263.227526 | Rh −0.081000 −0.296782 −0.126953  Cl −0.477917 −0.421104 2.199729  Cl −0.221082 −0.374741 −2.527061  P −1.077924 1.938544 −0.199992  N −0.487270 −3.288433 −0.240540  N 1.641657 −2.843821 −0.553314  C 0.441845 −2.294336 −0.248220  C 0.066645 −4.590487 −0.691824  H −0.317711 −4.825917 −1.697274  H −0.237514 −5.392401 −0.004293  C 1.573874 −4.324249 −0.687208  H 2.090737 −4.794419 0.165871  H 2.071748 −4.644314 −1.612431  C −1.873802 −3.242640 0.156830  C −2.187479 −3.521536 1.509464  C −1.114511 −3.805088 2.528961  H −1.563728 −4.106087 3.485389  H −0.498851 −2.909530 2.702007  H −0.443776 −4.616149 2.201855  C −3.534998 −3.552509 1.886349  H −3.780206 −3.751846 2.933747  C −4.570993 −3.354578 0.961793  C −6.014470 −3.362905 1.406553  H −6.697388 −3.497519 0.555397  H −6.280677 −2.413846 1.901040  H −6.207513 −4.168806 2.130852  C −4.225966 −3.154386 −0.379196  H −5.017878 −3.044846 −1.126236  C −2.890320 −3.113848 −0.814096  C −2.592932 −3.002129 −2.287914  H −3.463535 −2.602300 −2.826137  H −2.374881 −3.996004 −2.716629  H −1.736097 −2.345886 −2.494002  C 2.959347 −2.266298 −0.479471  C 3.658149 −1.996756 −1.673954  C 3.011591 −2.169514 −3.026008  H 3.637110 −1.722139 −3.811218  H 2.015426 −1.702241 −3.060798  H 2.883586 −3.235702 −3.281909  C 4.985055 −1.553834 −1.572194  H 5.532175 −1.324359 −2.491827  C 5.627129 −1.400489 −0.336637  C 7.039807 −0.875053 −0.253840  H 7.607088 −1.366053 0.551087  H 7.036775 0.206441 −0.037855 |

**Table S1.** *Cont.*

| **Rh-I-IId** | |
| --- | --- |
| 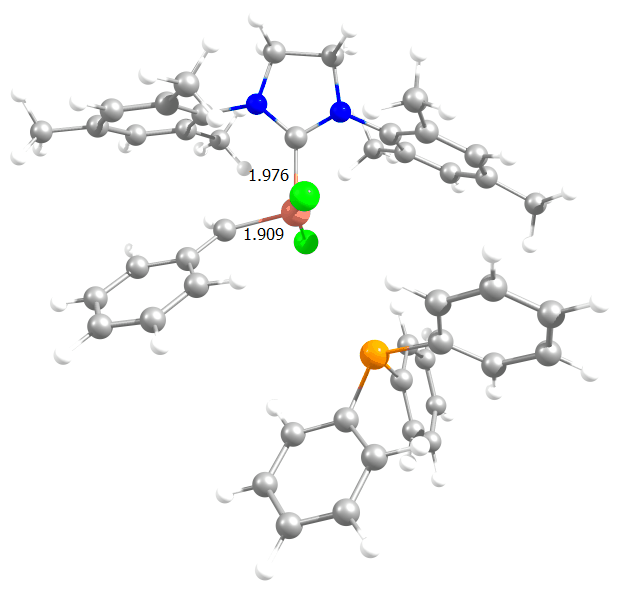  Zero-point correction= 0.778221 (Hartree/Particle)  Thermal correction to Energy= 0.831605  Thermal correction to Enthalpy= 0.832550  Thermal correction to Gibbs Free Energy= 0.681747  Sum of electronic and Zero-point Energies= −3263.111957  Sum of electronic and thermal Energies= −3263.058573  Sum of electronic and thermal Enthalpies= −3263.057628  Sum of electronic and thermal Free Energies= −3263.208431 | Rh −0.985901 −0.246268 0.169513  Cl −0.577599 0.144601 −2.134692  Cl −0.673716 −0.639660 2.461642  P 3.076358 0.935030 0.129291  N −2.099520 −2.870218 −0.447574  N −3.738636 −1.394354 −0.365180  C −2.404522 −1.568317 −0.211365  C −3.276347 −3.658189 −0.886981  H −3.371257 −4.569729 −0.279396  H −3.156331 −3.952439 −1.941408  C −4.436259 −2.669687 −0.679470  H −5.058498 −2.544593 −1.576954  H −5.092655 −2.951711 0.159324  C −0.798653 −3.482494 −0.320704  C 0.026702 −3.627351 −1.458786  C −0.436584 −3.245702 −2.841893  H 0.414463 −3.214328 −3.535633  H −0.920617 −2.259406 −2.859123  H −1.149465 −3.992114 −3.233731  C 1.294592 −4.200279 −1.280792  H 1.956298 −4.281805 −2.147561  C 1.730357 −4.674856 −0.037432  C 3.125818 −5.220527 0.144108  H 3.144667 −6.048212 0.868795  H 3.798389 −4.434204 0.526363  H 3.546556 −5.580828 −0.805481  C 0.837948 −4.617825 1.042087  H 1.137421 −5.034070 2.008410  C −0.432506 −4.040053 0.927254  C −1.366780 −4.033196 2.110012  H −0.982980 −4.690652 2.902414  H −2.375839 −4.383015 1.840712  H −1.461236 −3.017068 2.524546  C −4.541717 −0.232185 −0.094030  C −5.041742 −0.035872 1.209913  C −4.630701 −0.926627 2.356991  H −5.123467 −0.609962 3.286347  H −3.540611 −0.897484 2.521434  H −4.901166 −1.980691 2.181896  C −5.908857 1.046032 1.425609  H −6.298060 1.213019 2.434384  C −6.286766 1.912007 0.390958  C −7.236891 3.059206 0.641823  H −8.208060 2.884537 0.150378  H −6.836524 4.003389 0.24160 |

**Table S1.** *Cont.*

| **Rh-I-IIId** | |
| --- | --- |
| 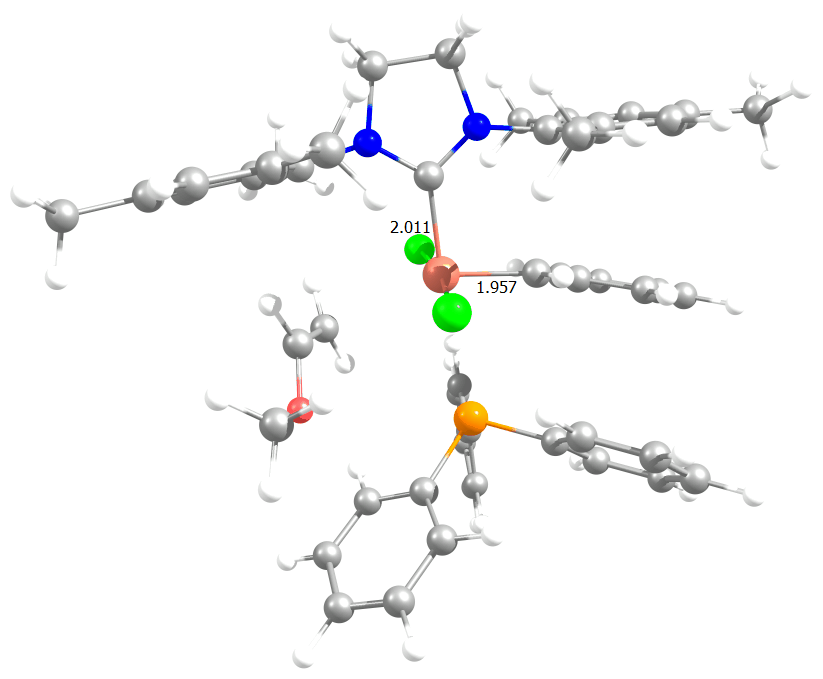  Zero-point correction= 0.863231 (Hartree/Particle)  Thermal correction to Energy= 0.923813  Thermal correction to Enthalpy= 0.924757  Thermal correction to Gibbs Free Energy= 0.763548  Sum of electronic and Zero-point Energies= −3456.200352  Sum of electronic and thermal Energies= −3456.139770  Sum of electronic and thermal Enthalpies= −3456.138825  Sum of electronic and thermal Free Energies= −3456.300034 | Rh 0.587676 0.181623 0.157655  Cl 0.455828 0.477751 2.543819  Cl 0.903182 −0.371580 −2.166776  P −1.811756 −1.932414 0.022525  N 3.332689 1.422582 0.407304  N 1.792691 2.961690 0.104301  C 1.996737 1.616604 0.177107  C 4.060451 2.695593 0.652835  H 4.994152 2.710809 0.073671  H 4.310425 2.776314 1.723234  C 3.050888 3.744223 0.204125  H 2.925276 4.563423 0.924823  H 3.291849 4.181938 −0.779230  C 4.170376 0.251609 0.355835  C 4.599475 −0.354618 1.556751  C 4.100053 0.102295 2.905496  H 4.238987 −0.688021 3.656605  H 3.035309 0.379602 2.886515  H 4.668094 0.981690 3.255687  C 5.546137 −1.387928 1.476244  H 5.864739 −1.875495 2.402363  C 6.103666 −1.797019 0.258356  C 7.102365 −2.928526 0.202598  H 7.762978 −2.840055 −0.672171  H 6.590041 −3.902679 0.131399  H 7.729440 −2.956767 1.105817  C 5.695623 −1.134897 −0.908807  H 6.140267 −1.415772 −1.868249  C 4.744464 −0.104422 −0.887709  C 4.367715 0.605513 −2.162106  H 4.972727 0.237202 −3.002143  H 4.529255 1.693117 −2.083127  H 3.302931 0.449549 −2.401363  C 0.632364 3.755831 −0.212922  C 0.350311 4.038670 −1.568095  C 1.102182 3.368932 −2.690850  H 0.707662 3.693498 −3.663765  H 1.029403 2.270440 −2.637758  H 2.175899 3.621185 −2.671508  C −0.660894 4.966524 −1.848955  H −0.899181 5.179435 −2.895576  C −1.361966 5.635168 −0.835403  C −2.476485 6.596756 −1.166771  H −2.518525 7.430099 −0.449544  H −3.451967 6.083266 −1.127960 |

**Table S1.** *Cont.*

| **Rh-I+** | |
| --- | --- |
| 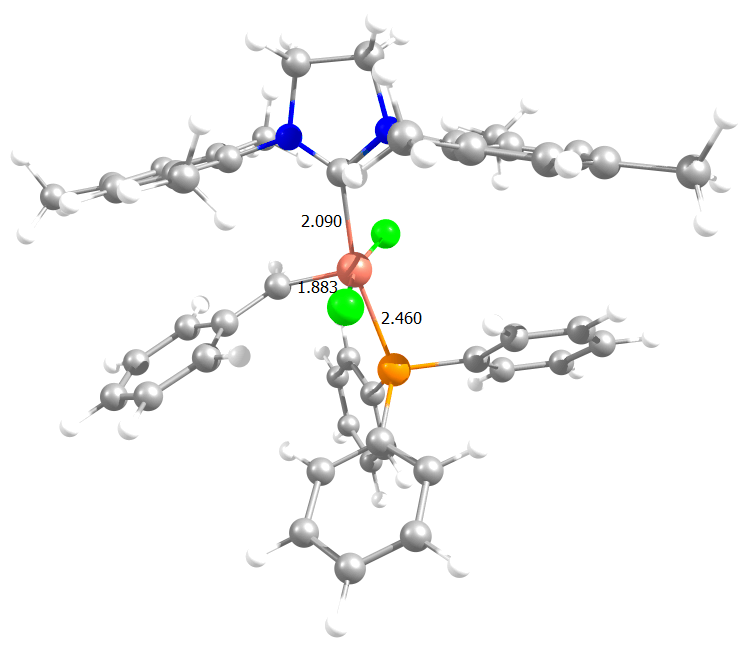  Zero-point correction= 0.783437 (Hartree/Particle)  Thermal correction to Energy= 0.837957  Thermal correction to Enthalpy= 0.838901  Thermal correction to Gibbs Free Energy= 0.690101  Sum of electronic and Zero-point Energies= −3262.949401  Sum of electronic and thermal Energies= −3262.894881  Sum of electronic and thermal Enthalpies= −3262.893937  Sum of electronic and thermal Free Energies= −3263.042738 | Rh −0.050729 −0.256585 −0.163370  Cl −0.420070 −0.333458 2.163734  Cl −0.269710 −0.352502 −2.529691  P −0.883114 2.058449 −0.196842  N −0.830600 −3.209052 −0.202109  N 1.342647 −2.993335 −0.479954  C 0.189592 −2.331052 −0.237308  C −0.391926 −4.600158 −0.506859  H −0.851836 −4.924938 −1.452237  H −0.722444 −5.279061 0.290770  C 1.134806 −4.465922 −0.595890  H 1.663259 −4.977904 0.222731  H 1.541668 −4.827425 −1.550026  C −2.221477 −2.997416 0.131642  C −2.620360 −3.208088 1.476726  C −1.637647 −3.588277 2.556279  H −2.113019 −3.528544 3.544453  H −0.759242 −2.929975 2.563613  H −1.287797 −4.627290 2.429356  C −3.980747 −3.109955 1.782305  H −4.294763 −3.257033 2.819718  C −4.952803 −2.868860 0.796845  C −6.417598 −2.820202 1.160204  H −7.033377 −2.479053 0.316482  H −6.598805 −2.149347 2.014142  H −6.782232 −3.817567 1.454635  C −4.525899 −2.720914 −0.525425  H −5.268048 −2.563750 −1.313379  C −3.170262 −2.795282 −0.891662  C −2.805989 −2.689472 −2.351880  H −2.919386 −1.655550 −2.711314  H −3.470428 −3.326313 −2.954993  H −1.766527 −2.974527 −2.552055  C 2.698425 −2.517287 −0.426630  C 3.408398 −2.319100 −1.634693  C 2.745526 −2.477001 −2.979860  H 3.372511 −2.048398 −3.773414  H 1.758686 −1.991643 −3.011566  H 2.598618 −3.541928 −3.227940  C 4.768358 −1.988935 −1.549556  H 5.325665 −1.825768 −2.476516  C 5.438662 −1.895263 −0.320892  C 6.917588 −1.604862 −0.263801  H 7.491501 −2.540258 −0.156861  H 7.172279 −0.970212 0.597505 |

**Table S1.** *Cont.*

| **Rh-I-II+** | |
| --- | --- |
| 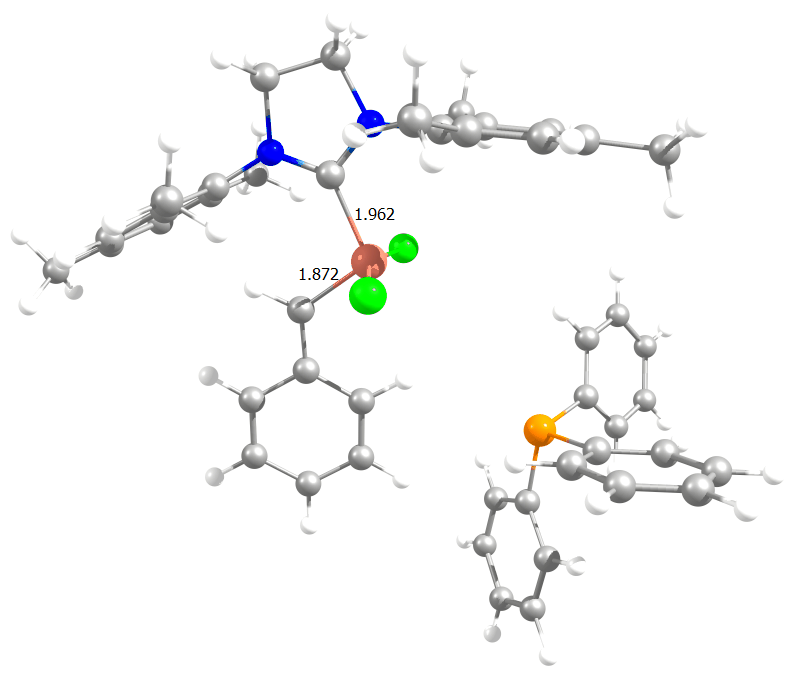  Zero-point correction= 0.780723 (Hartree/Particle)  Thermal correction to Energy= 0.835908  Thermal correction to Enthalpy= 0.836852  Thermal correction to Gibbs Free Energy= 0.679724  Sum of electronic and Zero-point Energies= −3262.913128  Sum of electronic and thermal Energies= −3262.857944  Sum of electronic and thermal Enthalpies= −3262.857000  Sum of electronic and thermal Free Energies= −3263.014127 | Rh −1.517368 −0.193701 −0.112379  Cl −1.324664 −0.473538 −2.391064  Cl −0.906156 −0.190625 2.106926  P 3.929997 0.537466 0.112358  N −3.239060 −2.418593 0.209903  N −4.499624 −0.596888 0.297493  C −3.252012 −1.074666 0.142879  C −4.597047 −2.975932 0.434463  H −4.609874 −3.548104 1.373093  H −4.860392 −3.649456 −0.393421  C −5.478057 −1.711112 0.487450  H −6.229273 −1.675513 −0.314001  H −5.990392 −1.584638 1.451184  C −2.076434 −3.264953 0.050842  C −1.767405 −3.769563 −1.236587  C −2.652568 −3.545047 −2.438000  H −2.051606 −3.415397 −3.347977  H −3.293868 −2.661673 −2.339521  H −3.298835 −4.425603 −2.595914  C −0.632114 −4.577457 −1.364540  H −0.368891 −4.953920 −2.356821  C 0.162080 −4.930966 −0.263632  C 1.408235 −5.759987 −0.443771  H 1.643476 −6.337915 0.460935  H 2.274457 −5.111480 −0.655414  H 1.310231 −6.458584 −1.286820  C −0.235870 −4.493676 1.007224  H 0.336509 −4.807454 1.884552  C −1.362657 −3.683925 1.200076  C −1.820324 −3.380335 2.605406  H −0.964684 −3.165947 3.259967  H −2.339186 −4.259259 3.024828  H −2.498881 −2.521720 2.659355  C −4.997537 0.754638 0.262239  C −5.136457 1.464999 1.474842  C −4.690884 0.882320 2.793824  H −4.817805 1.615658 3.600784  H −3.632528 0.579538 2.772447  H −5.280338 −0.008178 3.066917  C −5.690844 2.751534 1.415177  H −5.800721 3.316688 2.344831  C −6.118576 3.325805 0.207972  C −6.762936 4.689979 0.186992  H −6.633267 5.185831 −0.785258  H −6.352522 5.343977 0.969521 |

**Table S1.** *Cont.*

| **Rh-I+t** | |
| --- | --- |
| 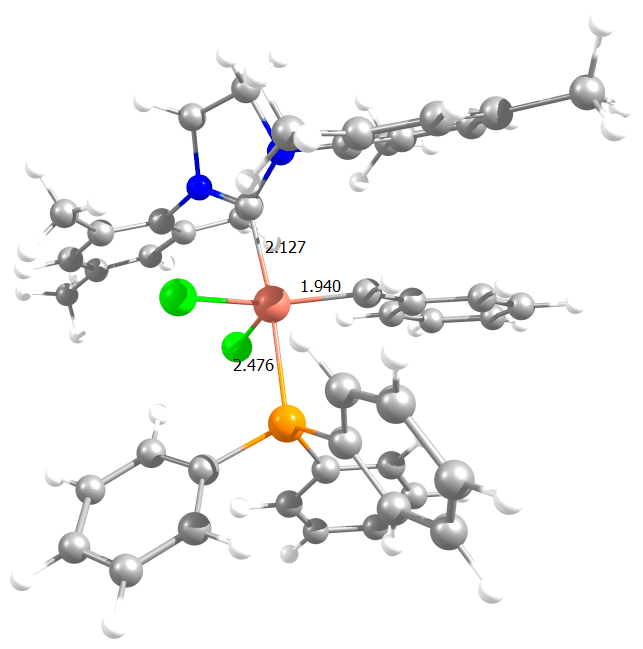  Zero-point correction= 0.781943 (Hartree/Particle)  Thermal correction to Energy= 0.837034  Thermal correction to Enthalpy= 0.837978  Thermal correction to Gibbs Free Energy= 0.685530  Sum of electronic and Zero-point Energies= −3262.899720  Sum of electronic and thermal Energies= −3262.844629  Sum of electronic and thermal Enthalpies= −3262.843685  Sum of electronic and thermal Free Energies= −3262.996133 | Rh −0.234067 −0.100321 −0.197577  Cl −1.778818 0.767088 1.439337  Cl −0.979925 0.342182 −2.428167  P 0.686361 2.197519 −0.243629  N −2.413549 −2.339734 −0.511378  N −0.384007 −3.086925 −0.913710  C −1.103844 −2.021913 −0.475330  C −2.652171 −3.727367 −1.003816  H −3.325451 −3.696124 −1.871148  H −3.136556 −4.315899 −0.210131  C −1.243388 −4.221735 −1.353546  H −0.959070 −5.139256 −0.819020  H −1.105615 −4.392915 −2.431394  C −3.573576 −1.634907 −0.011969  C −3.883752 −1.744742 1.362771  C −2.980936 −2.476373 2.324249  H −3.443247 −2.533126 3.318744  H −2.016753 −1.955723 2.429859  H −2.769418 −3.507082 1.996746  C −5.059375 −1.146805 1.824439  H −5.300367 −1.216876 2.888987  C −5.935970 −0.466365 0.964021  C −7.178386 0.202103 1.499753  H −7.901687 0.410604 0.699188  H −6.929708 1.162319 1.981218  H −7.675428 −0.421163 2.257884  C −5.619331 −0.423924 −0.397458  H −6.304464 0.073033 −1.090589  C −4.458141 −1.019271 −0.917796  C −4.238484 −1.027460 −2.410984  H −4.543452 −0.069150 −2.854813  H −4.853701 −1.810465 −2.886852  H −3.188484 −1.195323 −2.677896  C 1.039208 −3.286835 −0.919695  C 1.778315 −3.057219 −2.106280  C 1.124077 −2.545776 −3.363872  H 1.880224 −2.199287 −4.081256  H 0.420950 −1.723438 −3.168648  H 0.555534 −3.349138 −3.862247  C 3.145273 −3.363624 −2.098846  H 3.726295 −3.179460 −3.007141  C 3.779957 −3.934920 −0.983422  C 5.241566 −4.306243 −1.035153  H 5.388592 −5.221281 −1.631941  H 5.646883 −4.498893 −0.032276 |
| **Rh-IId** | |
| 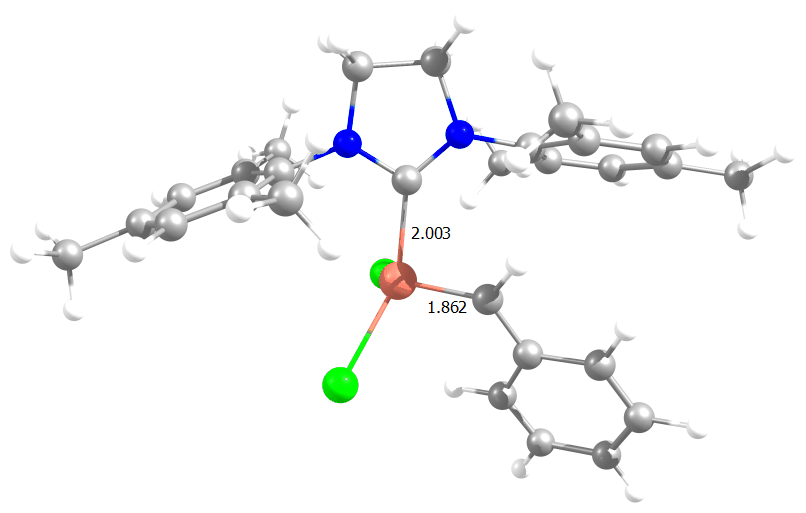  Zero-point correction= 0.513724 (Hartree/Particle)  Thermal correction to Energy= 0.550901  Thermal correction to Enthalpy= 0.551845  Thermal correction to Gibbs Free Energy= 0.439856  Sum of electronic and Zero-point Energies= −2226.842365  Sum of electronic and thermal Energies= −2226.805188  Sum of electronic and thermal Enthalpies= −2226.804244  Sum of electronic and thermal Free Energies= −2226.916232 | Rh −0.450891 0.892069 −0.009599  Cl −0.651052 1.048563 2.383255  Cl −1.371397 2.960110 −0.610706  N −1.719370 −1.692714 −0.060106  N 0.441263 −2.104507 −0.104966  C −0.489865 −1.110293 −0.056844  C −1.665718 −3.162152 −0.230512  H −2.013315 −3.434827 −1.241995  H −2.312510 −3.664118 0.502550  C −0.174713 −3.455868 −0.017494  H 0.032008 −3.890121 0.974713  H 0.246966 −4.124233 −0.781358  C −2.974609 −0.989603 −0.149204  C −3.780632 −0.897709 1.006962  C −3.313830 −1.430816 2.337031  H −4.084875 −1.278495 3.104647  H −2.396813 −0.909951 2.658298  H −3.089870 −2.509414 2.297773  C −5.023520 −0.261229 0.893843  H −5.647893 −0.170835 1.787418  C −5.481383 0.267758 −0.320275  C −6.801681 0.995679 −0.399190  H −7.262435 0.890357 −1.392391  H −6.660848 2.073838 −0.215478  H −7.512968 0.624240 0.352879  C −4.666945 0.132599 −1.452442  H −5.009706 0.532766 −2.410996  C −3.415987 −0.495031 −1.396752  C −2.567751 −0.595599 −2.640983  H −3.170720 −0.390710 −3.536399  H −2.109790 −1.589849 −2.756508  H −1.747968 0.141822 −2.611006  C 1.873795 −2.007239 −0.019892  C 2.628922 −2.189548 −1.199288  C 1.959707 −2.396541 −2.537636  H 2.696723 −2.351221 −3.351152 |

**Table S1.** *Cont.*

| **Rh-II-IIId** | |
| --- | --- |
| 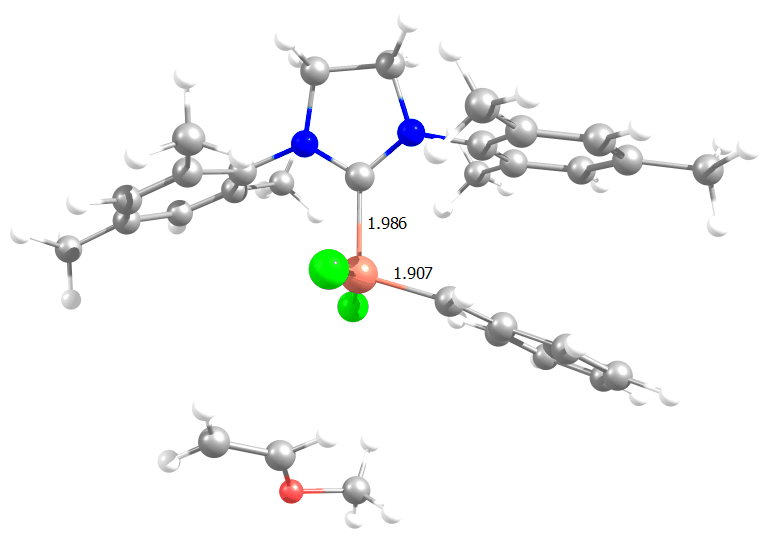  Zero-point correction= 0.596176 (Hartree/Particle)  Thermal correction to Energy= 0.639645  Thermal correction to Enthalpy= 0.640589  Thermal correction to Gibbs Free Energy= 0.513000  Sum of electronic and Zero-point Energies= −2419.937562  Sum of electronic and thermal Energies= −2419.894093  Sum of electronic and thermal Enthalpies= −2419.893149  Sum of electronic and thermal Free Energies= −2420.020738 | Rh −0.378117 0.515141 −0.332847  Cl −0.704031 1.433532 1.798442  Cl −0.689001 0.221004 −2.660956  N −1.549371 −2.109715 0.285621  N 0.636092 −2.306693 0.055918  C −0.396186 −1.431155 0.061232  C −1.337479 −3.577051 0.344874  H −1.778370 −4.053883 −0.544870  H −1.820028 −3.994601 1.240008  C 0.195480 −3.690340 0.374731  H 0.581579 −3.984922 1.364196  H 0.589253 −4.393401 −0.372414  C −2.876945 −1.552995 0.393405  C −3.352305 −1.186796 1.673682  C −2.510459 −1.356407 2.912832  H −3.120180 −1.200109 3.813354  H −1.686147 −0.626187 2.927860  H −2.067840 −2.363240 2.974733  C −4.647052 −0.661928 1.770109  H −5.016224 −0.357228 2.753620  C −5.477890 −0.521768 0.650032  C −6.845107 0.105824 0.774176  H −7.530156 −0.261150 −0.003835  H −6.779280 1.201212 0.665417  H −7.295471 −0.098747 1.756585  C −5.000192 −0.968454 −0.588346  H −5.647610 −0.903992 −1.467634  C −3.714855 −1.506846 −0.743764  C −3.287299 −2.055151 −2.081890  H −3.969873 −1.712625 −2.871616  H −3.314934 −3.158756 −2.083266  H −2.273223 −1.730978 −2.354655  C 2.045406 −2.039786 −0.042815  C 2.688178 −2.218169 −1.284541  C 1.912344 −2.556851 −2.532767  H 2.586778 −2.633629 −3.396687 |
| **Rh-II+** | |
| 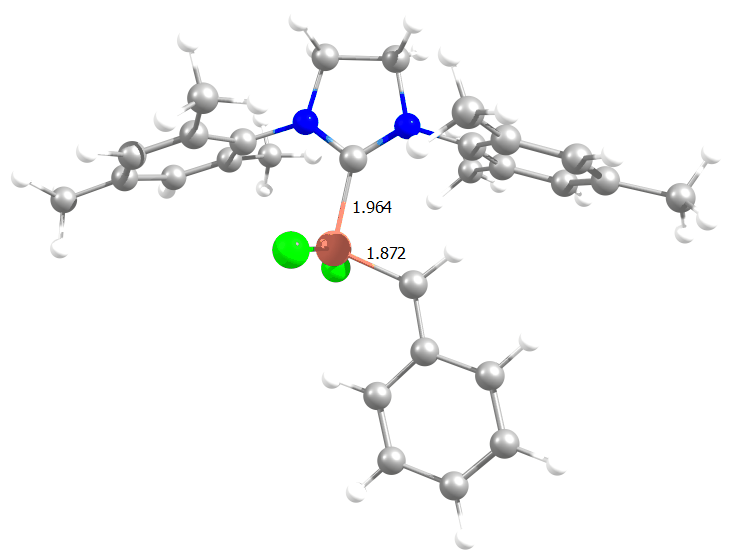  Zero-point correction= 0.515783 (Hartree/Particle)  Thermal correction to Energy= 0.552809  Thermal correction to Enthalpy= 0.553753  Thermal correction to Gibbs Free Energy= 0.442459  Sum of electronic and Zero-point Energies= −2226.633557  Sum of electronic and thermal Energies= −2226.596532  Sum of electronic and thermal Enthalpies= −2226.595587  Sum of electronic and thermal Free Energies= −2226.706881 | Rh −0.349358 0.819152 −0.055858  Cl −0.597322 1.240220 2.193698  Cl −0.690078 1.077672 −2.317079  N −1.734351 −1.645826 0.055610  N 0.433857 −2.112982 0.068078  C −0.489800 −1.137618 0.027995  C −1.724274 −3.129467 0.125509  H −2.252550 −3.542708 −0.745208  H −2.238059 −3.458093 1.040035  C −0.216667 −3.458257 0.128886  H 0.108782 −3.978167 1.040743  H 0.094455 −4.053216 −0.741031  C −2.960738 −0.877376 0.031997  C −3.568794 −0.511742 1.258232  C −3.054761 −0.974365 2.599168  H −3.177240 −0.189153 3.357250  H −1.994445 −1.251152 2.581738  H −3.635844 −1.848773 2.938864  C −4.749023 0.240407 1.201296  H −5.215566 0.549595 2.140696  C −5.357435 0.585407 −0.013661  C −6.601511 1.436535 −0.042809  H −7.228483 1.205801 −0.915453  H −6.336102 2.505194 −0.103498  H −7.205108 1.300813 0.865472  C −4.778945 0.114678 −1.201790  H −5.269027 0.326978 −2.155911  C −3.598947 −0.638226 −1.210613  C −3.110514 −1.233245 −2.507984  H −3.342114 −0.570031 −3.352033  H −3.623015 −2.192251 −2.698123  H −2.029349 −1.412825 −2.517993  C 1.872834 −2.037023 0.060454  C 2.551915 −2.124735 −1.174649  C 1.810186 −2.203074 −2.487246  H 2.515412 −2.204686 −3.328400 |

**Table S1.** *Cont.*

| **Rh-II-III+** | |
| --- | --- |
| 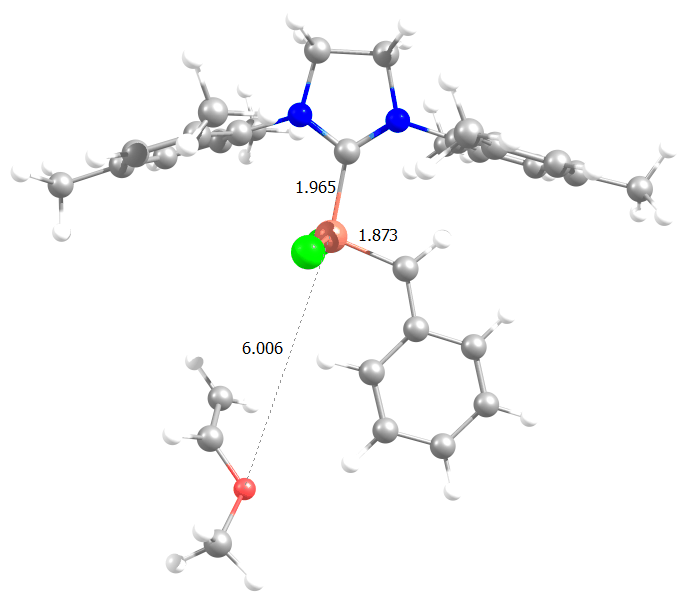  Zero-point correction= 0.597836 (Hartree/Particle)  Thermal correction to Energy= 0.641679  Thermal correction to Enthalpy= 0.642624  Thermal correction to Gibbs Free Energy= 0.510695  Sum of electronic and Zero-point Energies= −2419.735625  Sum of electronic and thermal Energies= −2419.691782  Sum of electronic and thermal Enthalpies= −2419.690837  Sum of electronic and thermal Free Energies= −2419.822766 | Rh 0.282139 0.039250 −0.121879  Cl 0.587040 −0.105473 −2.399544  Cl 0.819429 0.398292 2.088405  N 0.096040 −2.769462 0.220685  N −1.969710 −1.964686 0.255473  C −0.664843 −1.662930 0.133871  C −0.723905 −3.990730 0.425946  H −0.436743 −4.471295 1.372044  H −0.545247 −4.696512 −0.397627  C −2.162367 −3.435365 0.443187  H −2.784368 −3.828450 −0.373115  H −2.681388 −3.615746 1.394848  C 1.536947 −2.827608 0.102900  C 2.111504 −3.081821 −1.167324  C 1.285697 −3.382427 −2.393756  H 1.758859 −2.968959 −3.294388  H 0.271030 −2.972020 −2.338502  H 1.211204 −4.474276 −2.536524  C 3.507439 −3.137762 −1.251604  H 3.963986 −3.313291 −2.229706  C 4.331002 −2.999185 −0.124419  C 5.832804 −3.022629 −0.258281  H 6.317549 −3.331471 0.678236  H 6.214970 −2.019975 −0.512410  H 6.155662 −3.704785 −1.057678  C 3.719871 −2.844281 1.127270  H 4.343074 −2.789204 2.024135  C 2.328146 −2.783229 1.276628  C 1.735714 −2.770109 2.664122  H 2.333892 −2.143694 3.339556  H 1.737788 −3.792203 3.080214  H 0.708474 −2.389745 2.687224  C −3.133993 −1.116650 0.212774  C −3.639416 −0.587408 1.420252  C −2.940783 −0.809722 2.739413  H −3.492704 −0.323645 3.554298 |
| **RhIITZVP+t** | |
| 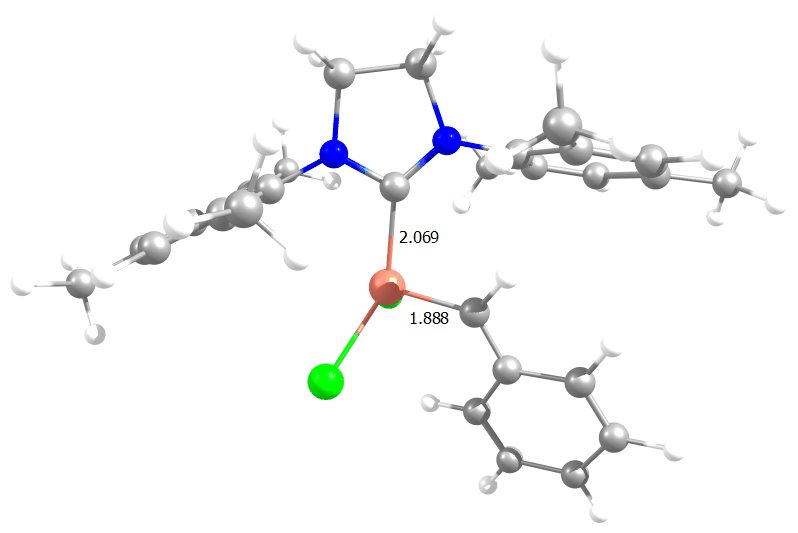  Zero-point correction= 0.514060 (Hartree/Particle)  Thermal correction to Energy= 0.551859  Thermal correction to Enthalpy= 0.552803  Thermal correction to Gibbs Free Energy= 0.437697  Sum of electronic and Zero-point Energies= −2226.599591  Sum of electronic and thermal Energies= −2226.561792  Sum of electronic and thermal Enthalpies= −2226.560848  Sum of electronic and thermal Free Energies= −2226.675954 | Rh −0.467156 0.785099 0.121855  Cl −0.473555 1.403997 2.355029  Cl −1.490187 2.503882 −1.005465  N −1.676678 −1.818297 −0.137112  N 0.490856 −2.230731 −0.131954  C −0.450866 −1.273032 −0.093328  C −1.637021 −3.303342 −0.217606  H −2.116014 −3.636396 −1.149893  H −2.184085 −3.740417 0.629382  C −0.121392 −3.596646 −0.176932  H 0.183959 −4.159448 0.716898  H 0.235198 −4.135073 −1.065434  C −2.901463 −1.052337 −0.153400  C −3.607730 −0.866268 1.058600  C −3.110683 −1.421594 2.370868  H −3.635745 −0.949380 3.211270  H −2.033989 −1.253425 2.513424  H −3.295500 −2.506790 2.442291  C −4.814309 −0.157968 1.004710  H −5.363333 0.011244 1.934872  C −5.340787 0.331073 −0.200902  C −6.628469 1.113559 −0.219328  H −7.164864 0.985816 −1.169999  H −6.425567 2.190868 −0.099493  H −7.295598 0.813078 0.600661  C −4.627066 0.091730 −1.385448  H −5.031107 0.452896 −2.334810  C −3.410972 −0.598996 −1.393998  C −2.676064 −0.827144 −2.692575  H −3.331871 −0.613180 −3.546537  H −2.318060 −1.862715 −2.795857  H −1.801117 −0.162387 −2.777194  C 1.920612 −2.083224 −0.030888  C 2.698984 −2.193195 −1.205205  C 2.073662 −2.416582 −2.562444  H 2.771895 −2.138923 −3.363227 |

**Table S1.** *Cont.*

| **RhIIITZVPd** | |
| --- | --- |
| 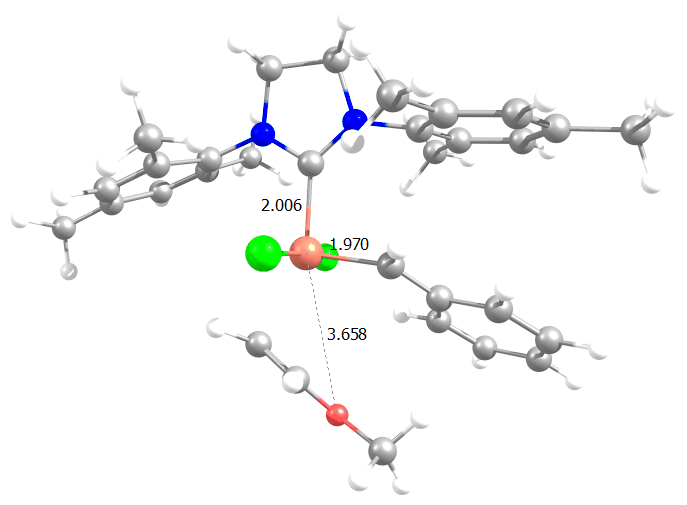  Zero-point correction= 0.597775 (Hartree/Particle)  Thermal correction to Energy= 0.640837  Thermal correction to Enthalpy= 0.641781  Thermal correction to Gibbs Free Energy= 0.517944  Sum of electronic and Zero-point Energies= −2419.945501  Sum of electronic and thermal Energies= −2419.902438  Sum of electronic and thermal Enthalpies= −2419.901494  Sum of electronic and thermal Free Energies= −2420.025331 | Rh −0.498155 0.756382 −0.244903  Cl −0.622926 1.198779 2.077436  Cl −0.706379 0.468594 −2.611890  N −1.945036 −1.726795 0.246972  N 0.191471 −2.236695 0.105725  C −0.694850 −1.215074 0.068623  C −1.953867 −3.207805 0.295416  H −2.407022 −3.607084 −0.626716  H −2.542129 −3.559789 1.154363  C −0.461510 −3.538417 0.411002  H −0.174387 −3.863705 1.424162  H −0.130309 −4.303523 −0.304404  C −3.194299 −1.003275 0.254519  C −3.759697 −0.638873 1.501266  C −3.114939 −1.019795 2.809036  H −3.702737 −0.632210 3.652418  H −2.095686 −0.614393 2.885057  H −3.056539 −2.115563 2.921316  C −4.977445 0.052817 1.497765  H −5.408368 0.355114 2.456690  C −5.662265 0.351500 0.311110  C −6.944547 1.148412 0.335598  H −7.571019 0.931070 −0.541587  H −6.732810 2.230768 0.328974  H −7.532864 0.937795 1.240990  C −5.124655 −0.114813 −0.893960  H −5.671857 0.053173 −1.826411  C −3.912352 −0.820482 −0.949637  C −3.481410 −1.439951 −2.255641  H −3.866510 −0.860805 −3.106184  H −3.894434 −2.460730 −2.341652  H −2.391790 −1.487012 −2.361648  C 1.629581 −2.238338 0.022095  C 2.228508 −2.573590 −1.209774  C 1.405539 −2.753130 −2.461747  H 2.055750 −2.953428 −3.324575 |
| **RhIII-IVTZVPd** | |
| 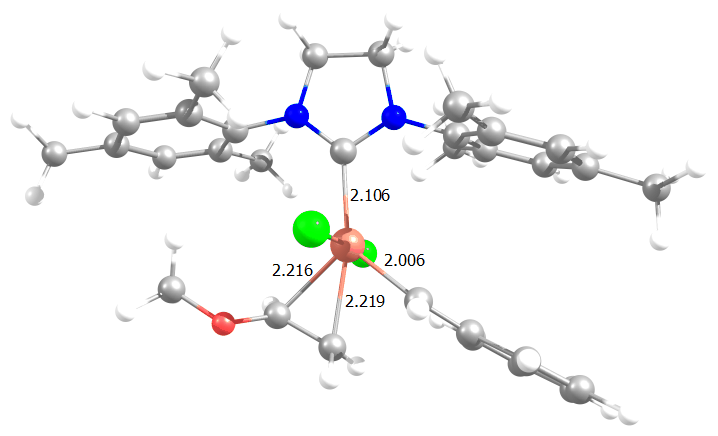  Zero-point correction= 0.598287 (Hartree/Particle)  Thermal correction to Energy= 0.640080  Thermal correction to Enthalpy= 0.641025  Thermal correction to Gibbs Free Energy= 0.521035  Sum of electronic and Zero-point Energies= −2419.933635  Sum of electronic and thermal Energies= −2419.891842  Sum of electronic and thermal Enthalpies= −2419.890898  Sum of electronic and thermal Free Energies= −2420.010888 | Rh 0.004304 −0.825789 −0.160380  Cl −0.285416 −0.939484 2.220144  Cl 0.617775 −0.708717 −2.470754  N 1.950225 1.646583 0.188062  N −0.174499 2.187357 0.103299  C 0.695477 1.150180 0.072811  C 1.966820 3.121114 0.403005  H 2.660984 3.599869 −0.301838  H 2.308139 3.340782 1.427496  C 0.502939 3.509074 0.167119  H 0.079177 4.113315 0.981036  H 0.352266 4.049863 −0.781004  C 3.215732 0.960137 0.222179  C 3.729151 0.506357 1.456613  C 2.940034 0.621390 2.735588  H 3.504599 0.194412 3.576095  H 1.969059 0.104975 2.668597  H 2.726743 1.674539 2.983091  C 5.022578 −0.039227 1.473698  H 5.424942 −0.396339 2.426512  C 5.817784 −0.111425 0.322553  C 7.202624 −0.713586 0.368439  H 7.900083 −0.167687 −0.284272  H 7.191775 −1.762019 0.026428  H 7.610361 −0.705291 1.389474  C 5.287833 0.382511 −0.878332  H 5.897044 0.352670 −1.786695  C 3.998938 0.930026 −0.954968  C 3.476718 1.463848 −2.264048  H 4.223537 1.332617 −3.059187  H 3.241607 2.539350 −2.203060  H 2.552557 0.940317 −2.557679  C −1.613627 2.153572 −0.007528  C −2.215305 2.205659 −1.283789  C −1.402127 2.257303 −2.552048  H −2.062646 2.356760 −3.424678 |

**Table S1.** *Cont.*

| **Rh-IIITZVP+** | |
| --- | --- |
| 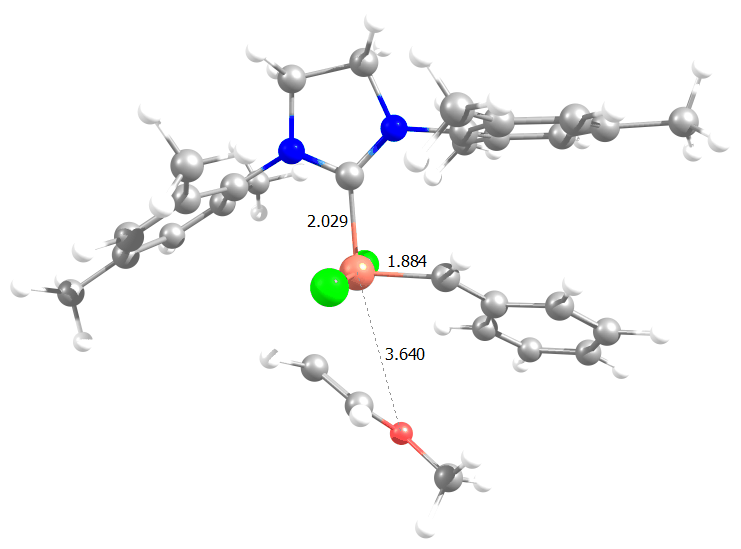  Zero-point correction= 0.601183 (Hartree/Particle)  Thermal correction to Energy= 0.643813  Thermal correction to Enthalpy= 0.644758  Thermal correction to Gibbs Free Energy= 0.523083  Sum of electronic and Zero-point Energies= −2419.756824  Sum of electronic and thermal Energies= −2419.714193  Sum of electronic and thermal Enthalpies= −2419.713249  Sum of electronic and thermal Free Energies= −2419.834923 | Rh −0.501072 0.686328 −0.150898  Cl −0.472279 0.956285 2.183602  Cl −0.844058 0.488522 −2.500227  N −1.878393 −1.832519 0.272625  N 0.277998 −2.296367 0.171101  C −0.646075 −1.321593 0.101697  C −1.856401 −3.305354 0.469193  H −2.415006 −3.795326 −0.341630  H −2.331699 −3.558771 1.427212  C −0.348474 −3.626255 0.441260  H 0.022640 −4.018022 1.399057  H −0.074342 −4.333746 −0.353441  C −3.114892 −1.082175 0.233317  C −3.679015 −0.611097 1.444410  C −3.121575 −0.967656 2.799835  H −3.251389 −0.138409 3.508396  H −2.054304 −1.211932 2.771653  H −3.669483 −1.834264 3.208562  C −4.864209 0.132853 1.368351  H −5.297986 0.516821 2.295987  C −5.523727 0.366880 0.153614  C −6.780766 1.199249 0.101989  H −7.438600 0.885696 −0.720862  H −6.538790 2.262924 −0.059868  H −7.346068 1.133034 1.042245  C −4.989316 −0.206305 −1.009501  H −5.522383 −0.089076 −1.957301  C −3.806994 −0.957909 −0.996960  C −3.379705 −1.681857 −2.249561  H −3.592677 −1.079911 −3.142986  H −3.948062 −2.623133 −2.345927  H −2.310251 −1.918722 −2.260617  C 1.707273 −2.215356 0.026177  C 2.272254 −2.432372 −1.254005  C 1.411309 −2.636652 −2.476306  H 2.034595 −2.742792 −3.373969 |
| **Rh-III-IVTZVP+** | |
| 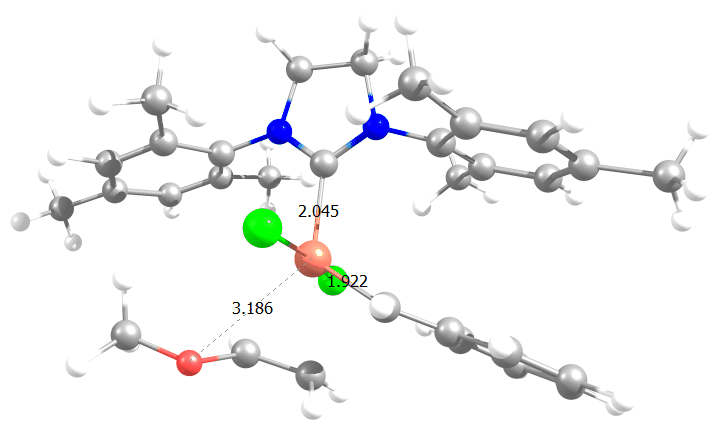  Zero-point correction= 0.601196 (Hartree/Particle)  Thermal correction to Energy= 0.642959  Thermal correction to Enthalpy= 0.643903  Thermal correction to Gibbs Free Energy= 0.524236  Sum of electronic and Zero-point Energies= −2419.745750  Sum of electronic and thermal Energies= −2419.703988  Sum of electronic and thermal Enthalpies= −2419.703044  Sum of electronic and thermal Free Energies= −2419.822711 | Rh 0.304969 −0.792121 −0.210013  Cl 0.431816 −1.220499 2.107874  Cl 0.624635 −0.390246 −2.518737  N 1.651357 1.835296 0.302740  N −0.528244 2.119016 0.075840  C 0.471930 1.224198 0.088973  C 1.487050 3.302402 0.503600  H 2.114048 3.848908 −0.213829  H 1.802657 3.570421 1.522712  C −0.020398 3.510128 0.266367  H −0.531666 3.975183 1.120400  H −0.234427 4.102911 −0.634445  C 2.955998 1.219400 0.394814  C 3.449946 0.825305 1.660281  C 2.711762 1.096801 2.947887  H 2.900307 0.302104 3.682337  H 1.627043 1.168736 2.812295  H 3.073089 2.040430 3.391788  C 4.723075 0.238101 1.713770  H 5.103535 −0.091932 2.684764  C 5.530338 0.097044 0.577337  C 6.914443 −0.496975 0.678193  H 7.234065 −0.945954 −0.272927  H 6.968763 −1.266216 1.461771  H 7.652831 0.280435 0.935019  C 5.039679 0.580871 −0.645058  H 5.674769 0.528944 −1.534382  C 3.771681 1.167243 −0.762679  C 3.350333 1.784976 −2.072669  H 4.010256 1.454655 −2.886219  H 3.426660 2.884804 −2.025207  H 2.319177 1.525091 −2.345072  C −1.947324 1.906014 −0.075928  C −2.540730 2.082889 −1.345268  C −1.731751 2.426653 −2.571553  H −2.328269 2.275312 −3.481259 |

**Table S1.** *Cont.*

| **Rh-IVTZVPd** | |
| --- | --- |
| 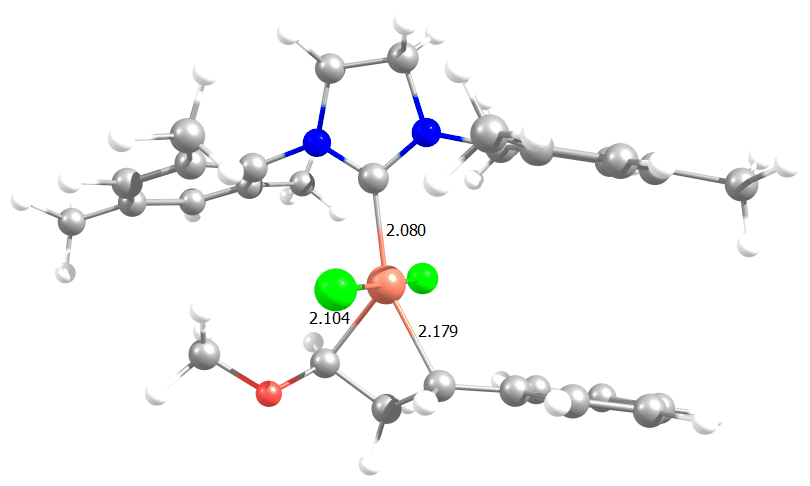  Zero-point correction= 0.600264 (Hartree/Particle)  Thermal correction to Energy= 0.642308  Thermal correction to Enthalpy= 0.643252  Thermal correction to Gibbs Free Energy= 0.521865  Sum of electronic and Zero-point Energies= −2419.964927  Sum of electronic and thermal Energies= −2419.922884  Sum of electronic and thermal Enthalpies= −2419.921940  Sum of electronic and thermal Free Energies= −2420.043327 | Rh 0.062627 −0.782367 −0.097343  Cl −0.349262 −0.854818 2.263451  Cl 0.501689 −0.661381 −2.442868  N 1.903076 1.720336 0.125901  N −0.232898 2.226434 0.004572  C 0.655419 1.207239 0.035450  C 1.894347 3.203392 0.272446  H 2.600405 3.658521 −0.435640  H 2.202979 3.475440 1.295076  C 0.432110 3.557137 −0.022414  H −0.014430 4.218825 0.732308  H 0.298697 4.018317 −1.014129  C 3.166722 1.033848 0.207038  C 3.652008 0.610416 1.464141  C 2.849813 0.788506 2.727849  H 3.402318 0.394562 3.592094  H 1.875807 0.275477 2.676380  H 2.643322 1.853344 2.926035  C 4.930875 0.035187 1.516756  H 5.311427 −0.300680 2.486099  C 5.739226 −0.093592 0.378852  C 7.105181 −0.732995 0.465952  H 7.773986 −0.361606 −0.324019  H 7.038254 −1.827927 0.351038  H 7.579848 −0.536911 1.438634  C 5.239716 0.377534 −0.842831  H 5.861661 0.305906 −1.740157  C 3.964409 0.950839 −0.955419  C 3.472530 1.453209 −2.289612  H 4.210892 1.243431 −3.075865  H 3.303516 2.542866 −2.279297  H 2.519438 0.973900 −2.565837  C −1.675911 2.160866 −0.049349  C −2.330965 2.163938 −1.300757  C −1.575268 2.201702 −2.604292  H −2.276073 2.208577 −3.450669 |
| **Rh-IV−VTZVPd** | |
| 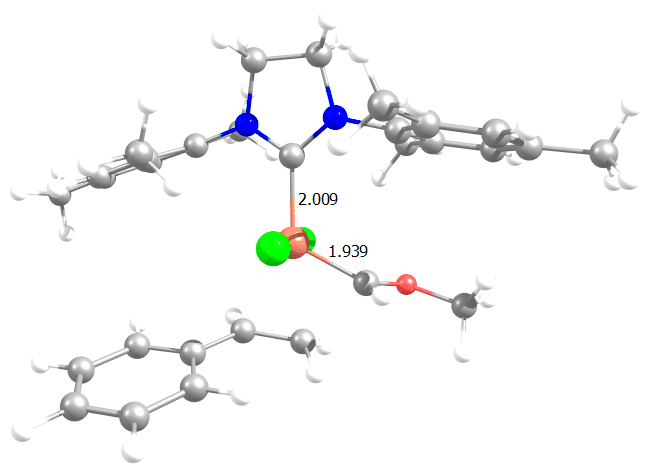  Zero-point correction= 0.597734 (Hartree/Particle)  Thermal correction to Energy= 0.639853  Thermal correction to Enthalpy= 0.640798  Thermal correction to Gibbs Free Energy= 0.519430  Sum of electronic and Zero-point Energies= −2419.943444  Sum of electronic and thermal Energies= −2419.901325  Sum of electronic and thermal Enthalpies= −2419.900381  Sum of electronic and thermal Free Energies= −2420.021748 | Rh −0.024494 0.551432 −0.484500  Cl −0.027329 1.658528 1.634658  Cl 0.128450 −0.530013 −2.601832  N −1.741580 −1.500234 0.941013  N 0.351802 −2.151854 0.730981  C −0.526176 −1.160495 0.439696  C −1.741628 −2.855105 1.551556  H −2.336538 −3.542654 0.927866  H −2.190793 −2.823426 2.554059  C −0.249213 −3.214981 1.574606  H 0.181800 −3.170628 2.587845  H −0.037183 −4.206851 1.151627  C −3.011855 −0.855527 0.727477  C −3.596148 −0.127367 1.787684  C −2.880778 0.074592 3.098799  H −3.469913 0.721335 3.763754  H −1.893674 0.536359 2.940634  H −2.720900 −0.881102 3.625667  C −4.877074 0.411085 1.593187  H −5.329782 0.987657 2.405502  C −5.594757 0.215444 0.405181  C −6.995357 0.761475 0.247206  H −7.739984 0.062850 0.664265  H −7.251713 0.916935 −0.810975  H −7.117626 1.718647 0.775621  C −4.993957 −0.536386 −0.613499  H −5.542424 −0.714114 −1.543708  C −3.712202 −1.091701 −0.476331  C −3.120104 −1.910632 −1.593760  H −3.866648 −2.082629 −2.381636  H −2.774695 −2.895230 −1.240150  H −2.247410 −1.411222 −2.046445  C 1.754554 −2.224011 0.396981  C 2.140620 −3.019292 −0.706401  C 1.126992 −3.734638 −1.562621  H 1.624332 −4.249449 −2.396173 |

**Table S1.** *Cont.*

| **Rh-IVTZVP+** | |
| --- | --- |
| 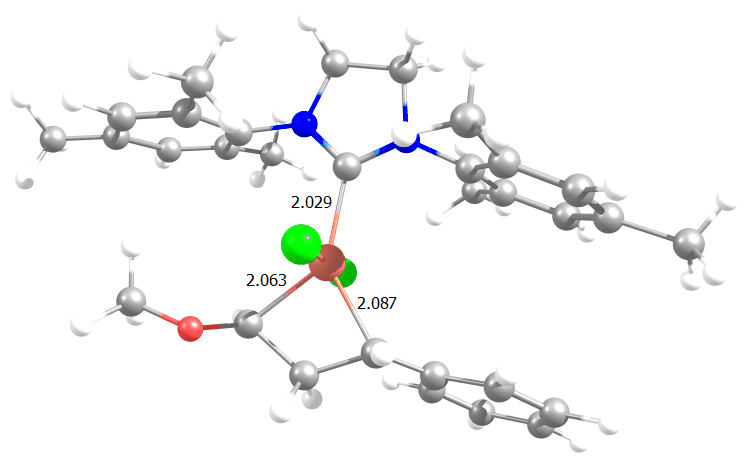  Zero-point correction= 0.602340 (Hartree/Particle)  Thermal correction to Energy= 0.644487  Thermal correction to Enthalpy= 0.645431  Thermal correction to Gibbs Free Energy= 0.524178  Sum of electronic and Zero-point Energies= −2419.753853  Sum of electronic and thermal Energies= −2419.711706  Sum of electronic and thermal Enthalpies= −2419.710762  Sum of electronic and thermal Free Energies= −2419.832015 | Rh 0.186384 −0.755017 −0.306649  Cl 0.116116 −1.354143 1.984454  Cl 0.382833 −0.124721 −2.574171  N 1.729373 1.694894 0.487524  N −0.440976 2.106046 0.322455  C 0.521345 1.179022 0.206632  C 1.613074 3.113503 0.937635  H 2.316269 3.739570 0.373069  H 1.864167 3.174948 2.007100  C 0.139993 3.442522 0.648481  H −0.385811 3.872119 1.511069  H 0.012340 4.113134 −0.214095  C 3.030664 1.063966 0.455432  C 3.576364 0.536204 1.646628  C 2.860185 0.597962 2.972983  H 3.195421 −0.213567 3.633134  H 1.771563 0.516360 2.873590  H 3.095540 1.545400 3.487974  C 4.879409 0.013567 1.595427  H 5.306449 −0.406099 2.510659  C 5.659913 0.058498 0.434134  C 7.063411 −0.496397 0.412636  H 7.760576 0.201043 −0.075159  H 7.109997 −1.442769 −0.150956  H 7.433074 −0.696023 1.427504  C 5.104723 0.653530 −0.711249  H 5.712403 0.743463 −1.616348  C 3.805914 1.179511 −0.725034  C 3.295374 1.892739 −1.951571  H 4.033395 1.838338 −2.762642  H 3.115481 2.961285 −1.746452  H 2.351569 1.462343 −2.315956  C −1.870072 1.986510 0.120608  C −2.422656 2.344846 −1.129974  C −1.578695 2.819393 −2.286589  H −2.132296 2.727637 −3.230783 |
| **Rh-IV−VTZVP+** | |
| 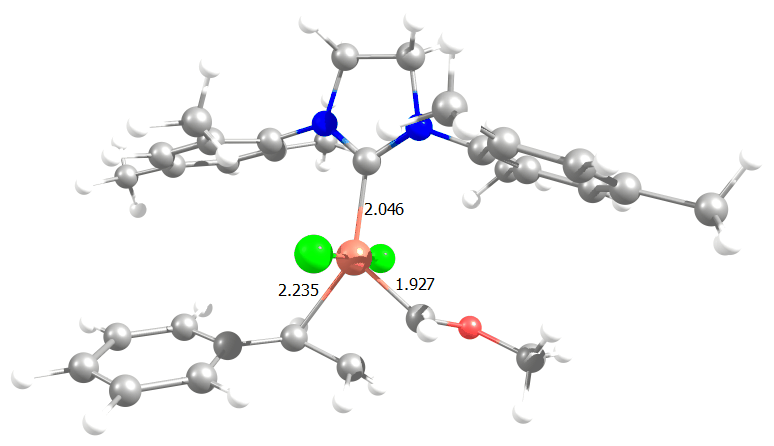  Zero-point correction= 0.601210 (Hartree/Particle)  Thermal correction to Energy= 0.643130  Thermal correction to Enthalpy= 0.644074  Thermal correction to Gibbs Free Energy= 0.523145  Sum of electronic and Zero-point Energies= −2419.751056  Sum of electronic and thermal Energies= −2419.709136  Sum of electronic and thermal Enthalpies= −2419.708192  Sum of electronic and thermal Free Energies= −2419.829121 | Rh −0.075444 0.637927 −0.425723  Cl −0.081072 1.530051 1.773219  Cl 0.012446 −0.292731 −2.590203  N −1.737569 −1.624895 0.725583  N 0.407618 −2.143665 0.587575  C −0.519125 −1.202222 0.351241  C −1.683472 −3.002149 1.300113  H −2.344336 −3.666328 0.725559  H −2.032228 −2.976963 2.341936  C −0.193670 −3.374032 1.175996  H 0.278689 −3.589274 2.144502  H −0.018162 −4.224735 0.502508  C −3.015198 −0.960714 0.607266  C −3.570283 −0.327541 1.741853  C −2.863236 −0.289755 3.073793  H −3.255273 0.525718 3.696416  H −1.781602 −0.140739 2.964917  H −3.032566 −1.226740 3.631961  C −4.850113 0.234650 1.618530  H −5.283503 0.737761 2.487779  C −5.599763 0.136370 0.437721  C −7.001361 0.692826 0.362466  H −7.729079 −0.034931 0.757726  H −7.295533 0.916366 −0.672774  H −7.105790 1.610082 0.959951  C −5.028728 −0.536990 −0.652352  H −5.606481 −0.648828 −1.574470  C −3.747774 −1.105716 −0.592824  C −3.203511 −1.866426 −1.775333  H −3.944843 −1.899936 −2.584586  H −2.960624 −2.908459 −1.510693  H −2.284307 −1.408812 −2.172260  C 1.822294 −2.091976 0.290461  C 2.292663 −2.676248 −0.909894  C 1.380350 −3.375299 −1.887264  H 1.815432 −3.366297 −2.895626 |

**Table S1.** *Cont.*

| **Rh-VTZVPd** | |
| --- | --- |
| 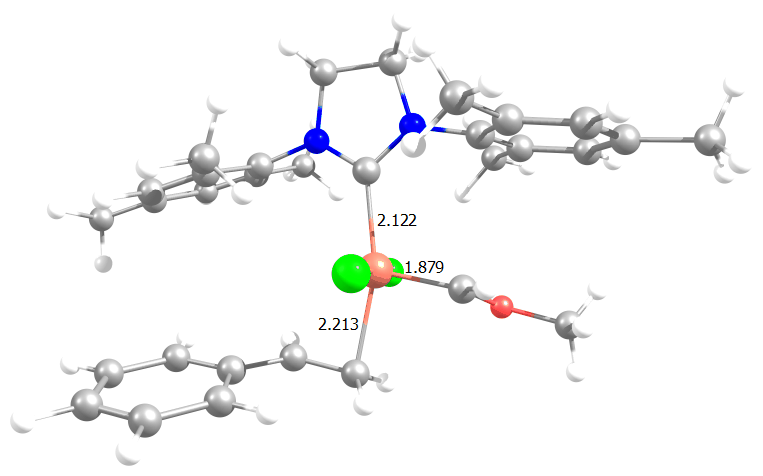  Zero-point correction= 0.597816 (Hartree/Particle)  Thermal correction to Energy= 0.640667  Thermal correction to Enthalpy= 0.641611  Thermal correction to Gibbs Free Energy= 0.519293  Sum of electronic and Zero-point Energies= −2419.954073  Sum of electronic and thermal Energies= −2419.911222  Sum of electronic and thermal Enthalpies= −2419.910278  Sum of electronic and thermal Free Energies= −2420.032596 | Rh 0.208588 −0.796872 −0.549303  Cl 0.100536 −1.851202 1.603836  Cl 0.203380 0.181379 −2.726942  N 1.772627 1.549319 0.775911  N −0.340313 2.075163 0.543238  C 0.573128 1.101119 0.325662  C 1.721334 2.950166 1.276358  H 2.292085 3.608189 0.600868  H 2.169203 3.014317 2.278350  C 0.218246 3.250344 1.267056  H −0.213926 3.299662 2.279517  H −0.035103 4.180136 0.738211  C 3.062596 0.918839 0.699594  C 3.569986 0.265308 1.844799  C 2.743231 0.115501 3.095992  H 3.305688 −0.428985 3.867033  H 1.810459 −0.433561 2.888383  H 2.463029 1.094911 3.517607  C 4.873577 −0.250535 1.789322  H 5.269298 −0.766877 2.669171  C 5.684293 −0.105323 0.654872  C 7.104690 −0.621129 0.647533  H 7.794636 0.119896 1.084978  H 7.455040 −0.828098 −0.374247  H 7.202066 −1.543590 1.238960  C 5.154166 0.567165 −0.455104  H 5.775145 0.704891 −1.346026  C 3.853688 1.096522 −0.457042  C 3.330174 1.826079 −1.667277  H 4.089207 1.850035 −2.461464  H 3.066745 2.870053 −1.430386  H 2.421392 1.348178 −2.069568  C −1.757421 2.063540 0.275600  C −2.232523 2.726145 −0.878250  C −1.292148 3.330884 −1.889779  H −1.857960 3.806360 −2.702950 |

| **Rh-V−VITZVPd** | |
| --- | --- |
| 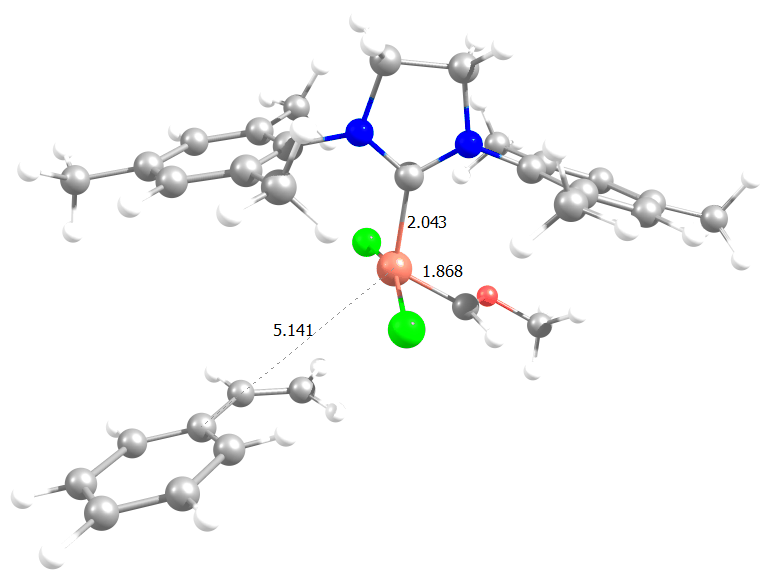  Zero-point correction= 0.596425 (Hartree/Particle)  Thermal correction to Energy= 0.639874  Thermal correction to Enthalpy= 0.640818  Thermal correction to Gibbs Free Energy= 0.512995  Sum of electronic and Zero-point Energies= −2419.947654  Sum of electronic and thermal Energies= −2419.904205  Sum of electronic and thermal Enthalpies= −2419.903261  Sum of electronic and thermal Free Energies= −2420.031084 | Rh 0.289028 −0.529290 −0.546118  Cl −0.293921 −1.676219 1.462607  Cl 0.311451 0.298799 −2.740632  N 2.295203 1.302534 0.848576  N 0.307182 2.227129 0.693368  C 1.034421 1.126431 0.390943  C 2.500650 2.646341 1.452917  H 3.186030 3.237786 0.823992  H 2.948543 2.549392 2.452368  C 1.076254 3.223652 1.485333  H 0.667297 3.286119 2.506236  H 1.002439 4.217186 1.020981  C 3.429258 0.430772 0.704003  C 3.776392 −0.416157 1.778603  C 2.901264 −0.530825 3.000515  H 3.353745 −1.207146 3.738823  H 1.903224 −0.918796 2.735446  H 2.748316 0.444899 3.489371  C 4.952573 −1.172961 1.669115  H 5.227676 −1.835552 2.495193  C 5.788208 −1.089127 0.545669  C 7.083397 −1.865371 0.483285  H 7.903137 −1.300259 0.957752  H 7.385209 −2.065985 −0.555123  H 7.004806 −2.827276 1.011265  C 5.404424 −0.243263 −0.504632  H 6.037253 −0.171694 −1.394493  C 4.232914 0.527203 −0.451889  C 3.836486 1.397857 −1.616878  H 4.625975 1.403030 −2.380886  H 3.656749 2.441116 −1.312531  H 2.906074 1.032972 −2.083623  C −1.081731 2.463662 0.382748  C −1.399660 3.184275 −0.789932  C −0.332479 3.659458 −1.742383  H −0.775042 4.271499 −2.540417 |

**Table S1.** *Cont.*

| **Rh-VTZVP+** | |
| --- | --- |
| 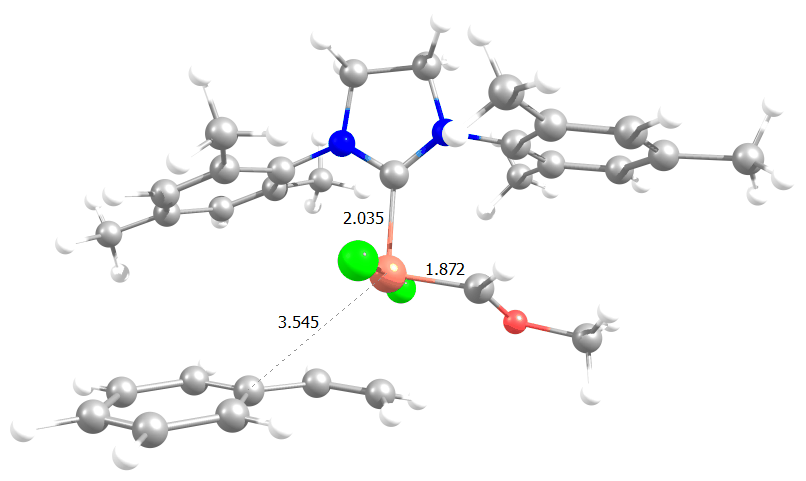  Zero-point correction= 0.600875 (Hartree/Particle)  Thermal correction to Energy= 0.643807  Thermal correction to Enthalpy= 0.644751  Thermal correction to Gibbs Free Energy= 0.521724  Sum of electronic and Zero-point Energies= −2419.764427  Sum of electronic and thermal Energies= −2419.721496  Sum of electronic and thermal Enthalpies= −2419.720551  Sum of electronic and thermal Free Energies= −2419.843578 | Rh 0.033553 −0.667475 −0.419953  Cl −0.218722 −1.511013 1.776479  Cl 0.062046 0.128009 −2.633074  N 1.951867 1.496349 0.661502  N −0.163235 2.103679 0.533022  C 0.711338 1.108179 0.307799  C 1.973796 2.913313 1.132121  H 2.561784 3.523321 0.430293  H 2.445663 2.970995 2.122174  C 0.482578 3.292546 1.152094  H 0.089971 3.433469 2.170092  H 0.258217 4.189861 0.559688  C 3.200134 0.790780 0.543846  C 3.728313 0.144567 1.688005  C 2.964509 0.079045 2.986952  H 3.500904 −0.539274 3.718587  H 1.955544 −0.341031 2.851957  H 2.843299 1.080132 3.432565  C 5.001819 −0.433372 1.586198  H 5.417501 −0.939534 2.462079  C 5.770948 −0.346341 0.414560  C 7.167401 −0.915569 0.363455  H 7.893822 −0.190502 0.766185  H 7.475900 −1.146538 −0.665793  H 7.255740 −1.829657 0.968100  C 5.221196 0.320626 −0.690624  H 5.811259 0.413469 −1.607036  C 3.947473 0.908374 −0.652336  C 3.415352 1.648304 −1.853511  H 4.131336 1.600902 −2.684494  H 3.246178 2.714195 −1.629823  H 2.455860 1.234570 −2.202429  C −1.586522 2.087821 0.277414  C −2.063462 2.646226 −0.936548  C −1.143418 3.278213 −1.950761  H −1.631368 3.334340 −2.932919 |
| **Rh-V−VITZVP+** | |
| 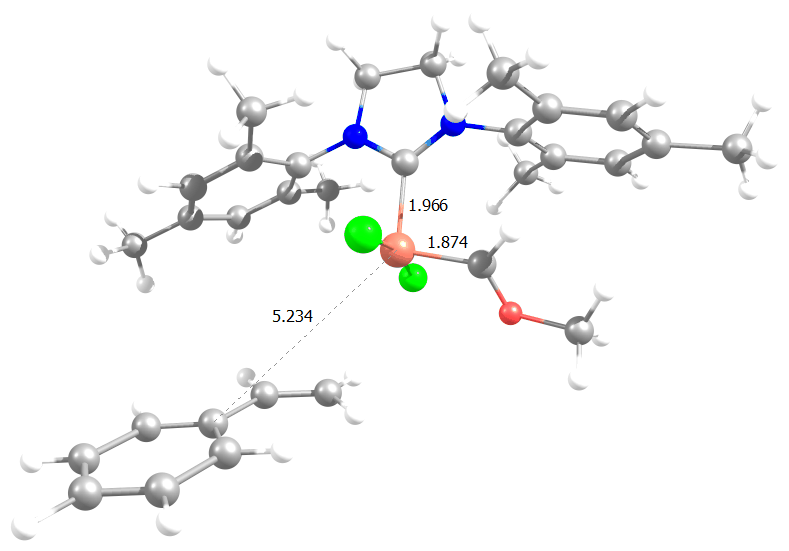  Zero-point correction= 0.598303 (Hartree/Particle)  Thermal correction to Energy= 0.641009  Thermal correction to Enthalpy= 0.641953  Thermal correction to Gibbs Free Energy= 0.515810  Sum of electronic and Zero-point Energies= −2419.746151  Sum of electronic and thermal Energies= −2419.703445  Sum of electronic and thermal Enthalpies= −2419.702501  Sum of electronic and thermal Free Energies= −2419.828644 | Rh 0.200440 −0.335712 −0.418595  Cl −0.455126 −1.152066 1.673355  Cl 0.441273 0.295951 −2.636572  N 2.574783 1.284717 0.563369  N 0.606352 2.300882 0.535261  C 1.264230 1.156496 0.293198  C 2.876693 2.647231 1.097837  H 3.679214 3.107032 0.505799  H 3.214619 2.565400 2.141124  C 1.527269 3.385330 0.964799  H 1.177662 3.818664 1.911678  H 1.546422 4.174732 0.198991  C 3.611272 0.287755 0.496403  C 3.913543 −0.465823 1.655752  C 3.125236 −0.313222 2.933621  H 3.454258 −1.048247 3.679571  H 2.044385 −0.454441 2.771121  H 3.260904 0.685250 3.379703  C 4.962234 −1.392969 1.576821  H 5.204090 −1.985797 2.463446  C 5.724374 −1.562285 0.408614  C 6.887226 −2.522965 0.377708  H 7.810903 −2.018305 0.705951  H 7.070826 −2.907421 −0.635461  H 6.728001 −3.376027 1.052438  C 5.401792 −0.783107 −0.712623  H 5.993265 −0.889736 −1.626289  C 4.362844 0.160731 −0.694018  C 4.075456 1.005893 −1.909270  H 4.795776 0.789550 −2.708827  H 4.148802 2.080878 −1.680176  H 3.061749 0.828097 −2.302046  C −0.813470 2.497680 0.335252  C −1.272677 3.034382 −0.895668  C −0.338487 3.505702 −1.982592  H −0.758713 3.299505 −2.976167 |

**Table S1.** *Cont.*

| **Rh-VITZVPd** | |
| --- | --- |
| 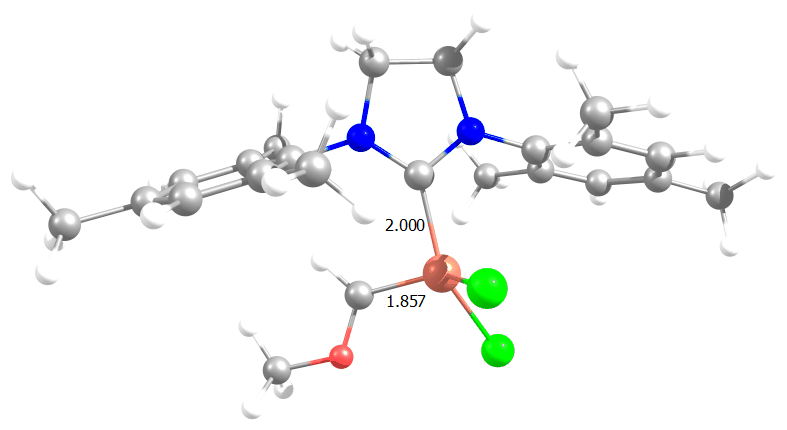  Zero-point correction= 0.466561 (Hartree/Particle)  Thermal correction to Energy= 0.502152  Thermal correction to Enthalpy= 0.503096  Thermal correction to Gibbs Free Energy= 0.393283  Sum of electronic and Zero-point Energies= −2110.339741  Sum of electronic and thermal Energies= −2110.304150  Sum of electronic and thermal Enthalpies= −2110.303206  Sum of electronic and thermal Free Energies= −2110.413019 | Rh −0.347373 1.146747 0.312040  Cl −1.642226 3.063011 −0.102473  Cl −0.601545 0.716360 2.660746  N 1.108528 −1.534122 −0.359243  N −1.090273 −1.571521 −0.305927  C −0.002843 −0.771245 −0.139566  C 0.776971 −2.966847 −0.580514  H 1.079898 −3.556259 0.300758  H 1.311861 −3.358153 −1.457437  C −0.745531 −2.934075 −0.770485  H −1.044919 −3.056036 −1.825726  H −1.269602 −3.693992 −0.174235  C 2.490560 −1.172683 −0.198894  C 3.266107 −0.950380 −1.358530  C 2.660479 −1.031014 −2.740688  H 3.327122 −0.573820 −3.485035  H 1.683503 −0.527911 −2.792692  H 2.498060 −2.077161 −3.050927  C 4.625372 −0.638689 −1.204488  H 5.227861 −0.451774 −2.098718  C 5.228155 −0.565661 0.058949  C 6.700460 −0.259267 0.203439  H 7.283242 −1.185045 0.342614  H 6.894923 0.380343 1.077105  H 7.096891 0.247131 −0.688359  C 4.434217 −0.820291 1.186044  H 4.888531 −0.780012 2.180528  C 3.071717 −1.140356 1.088444  C 2.271197 −1.446603 2.326460  H 2.916182 −1.431106 3.215594  H 1.806514 −2.444265 2.267305  H 1.452396 −0.724200 2.479879  C −2.462154 −1.128646 −0.325689  C −3.289704 −1.438863 0.775880  C −2.747001 −2.126874 2.001766  H −3.551014 −2.311462 2.727456 |
| **Rh-VITZVP+** | |
| 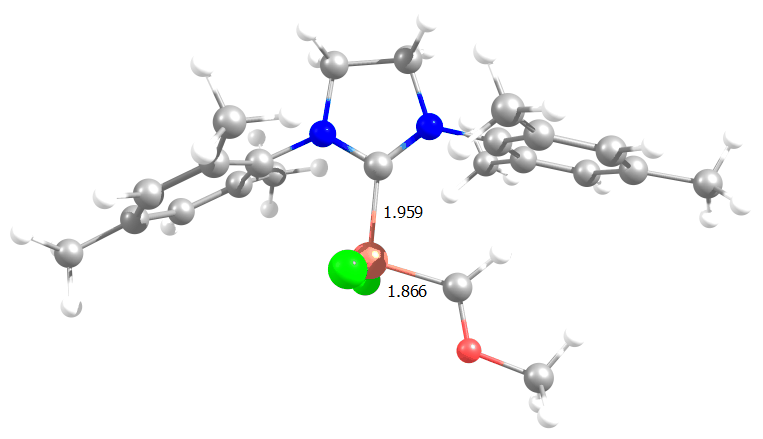  Zero-point correction= 0.468673 (Hartree/Particle)  Thermal correction to Energy= 0.504069  Thermal correction to Enthalpy= 0.505013  Thermal correction to Gibbs Free Energy= 0.397041  Sum of electronic and Zero-point Energies= −2110.134490  Sum of electronic and thermal Energies= −2110.099095  Sum of electronic and thermal Enthalpies= −2110.098150  Sum of electronic and thermal Free Energies= −2110.206122 | Rh −0.338997 1.126015 −0.015166  Cl −0.622318 1.377252 −2.295597  Cl −0.550147 1.459513 2.259660  N 0.982274 −1.610465 0.048974  N −1.234025 −1.537609 0.049706  C −0.101520 −0.818578 0.026229  C 0.584500 −3.051984 0.085346  H 1.001861 −3.524072 0.985456  H 0.987980 −3.565035 −0.798537  C −0.960857 −2.996907 0.095674  H −1.412657 −3.485487 −0.779035  H −1.398417 −3.426809 1.007817  C 2.372562 −1.236010 0.025656  C 3.025172 −1.112625 −1.221226  C 2.298166 −1.332728 −2.525964  H 2.965484 −1.141054 −3.376055  H 1.422473 −0.672485 −2.627563  H 1.939136 −2.370109 −2.620435  C 4.382002 −0.754803 −1.214355  H 4.900519 −0.653637 −2.171727  C 5.093099 −0.543452 −0.021840  C 6.565353 −0.214919 −0.044889  H 7.165037 −1.138846 0.001405  H 6.857113 0.403602 0.815665  H 6.850576 0.311249 −0.966649  C 4.406913 −0.689189 1.194716  H 4.945768 −0.536995 2.134037  C 3.050665 −1.044722 1.250246  C 2.351898 −1.193669 2.579738  H 3.044459 −0.981785 3.404499  H 1.971476 −2.217095 2.726730  H 1.494078 −0.509183 2.673140  C −2.563949 −0.966285 0.026843  C −3.228519 −0.713761 1.253666  C −2.662323 −1.119608 2.591240  H −2.909127 −0.377848 3.362504 |

**Table S1.** *Cont.*

| **Ru−I** | |
| --- | --- |
| 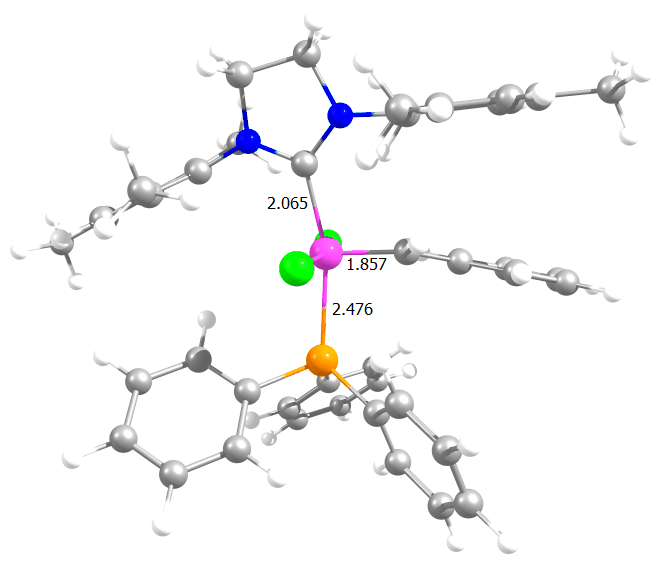  Zero-point correction= 0.781132 (Hartree/Particle)  Thermal correction to Energy= 0.835682  Thermal correction to Enthalpy= 0.836626  Thermal correction to Gibbs Free Energy= 0.687548  Sum of electronic and Zero-point Energies= −3247.502187  Sum of electronic and thermal Energies= −3247.447637  Sum of electronic and thermal Enthalpies= −3247.446692  Sum of electronic and thermal Free Energies= −3247.595770 | Ru 0.030857 −0.298599 −0.073068  Cl −0.257592 −0.346407 2.322858  Cl −0.182789 −0.446325 −2.507387  P −1.181151 1.858174 −0.180370  N −0.371079 −3.319889 −0.097146  N 1.756614 −2.857062 −0.382767  C 0.535895 −2.299634 −0.138810  C 0.216616 −4.631016 −0.463974  H −0.125733 −4.925800 −1.469651  H −0.097242 −5.404350 0.251753  C 1.718355 −4.341017 −0.421907  H 2.207040 −4.751358 0.478395  H 2.254156 −4.715963 −1.305179  C −1.781461 −3.268681 0.197357  C −2.193154 −3.450501 1.540620  C −1.198490 −3.650082 2.654160  H −1.713338 −3.939498 3.580886  H −0.634593 −2.722742 2.842334  H −0.469240 −4.438809 2.409188  C −3.565843 −3.475866 1.817351  H −3.887508 −3.599456 2.855727  C −4.531261 −3.375757 0.805271  C −6.004695 −3.380767 1.139031  H −6.616414 −3.618243 0.256648  H −6.328850 −2.395636 1.514282  H −6.236902 −4.116393 1.924053  C −4.088746 −3.277056 −0.518601  H −4.822745 −3.245651 −1.329451  C −2.724922 −3.240244 −0.853864  C −2.317912 −3.241492 −2.305812  H −3.140087 −2.874637 −2.935992  H −2.085612 −4.267750 −2.641003  H −1.440233 −2.607508 −2.495027  C 3.053693 −2.240742 −0.349865  C 3.726777 −2.001378 −1.566929  C 3.060896 −2.254666 −2.896067  H 3.699892 −1.906951 −3.719943  H 2.086936 −1.742141 −2.962617  H 2.871340 −3.329331 −3.058338  C 5.038753 −1.507358 −1.509564  H 5.565695 −1.310482 −2.448264  C 5.691685 −1.268873 −0.293199  C 7.095105 −0.712277 −0.256955  H 7.715937 −1.233476 0.487800  H 7.085045 0.355260 0.018616  H 7.588664 −0.799967 −1.235505  C 4.999646 −1.540750 0.895797  H 5.493521 −1.366198 1.856282  C 3.688488 −2.032377 0.895999  C 2.977079 −2.318402 2.193441  H 2.682699 −3.378049 2.271908  H 2.054663 −1.722326 2.288137  H 3.627014 −2.089648 3.049161  C 1.711351 0.454341 −0.309815  C 3.649881 1.955361 −0.207661  C 2.612813 1.286095 0.490556  C 4.558421 2.777324 0.459241  C 2.541259 1.455238 1.892996  C 4.473474 2.927244 1.848572  H 5.336682 3.298462 −0.103258  C 3.468430 2.256075 2.559468  H 1.752222 0.935627 2.441098  H 5.188612 3.562635 2.376594  H 3.406057 2.363425 3.645195  C −2.807451 1.607077 −1.044246  C −3.249898 2.449524 −2.075160  C −3.614338 0.528062 −0.643667  C −4.486331 2.222864 −2.689864  H −2.626324 3.282254 −2.404079  C −4.853600 0.311617 −1.252922  H −3.280168 −0.145401 0.150104  C −5.292767 1.157539 −2.277720  H −4.817690 2.883420 −3.494496  H −5.470660 −0.528897 −0.929555  H −6.258547 0.982675 −2.757467  C −0.402082 3.266532 −1.124988  C −0.581448 4.603128 −0.728677  C 0.354296 2.986158 −2.275714  C −0.004886 5.642087 −1.468437  H −1.168056 4.835939 0.161154  C 0.920397 4.029475 −3.015273  H 0.476019 1.948329 −2.598583  C 0.747036 5.358655 −2.612983  H −0.146019 6.676166 −1.145232 |

**Table S1.** *Cont.*

| **Ru−I** | |
| --- | --- |
|  | H 1.501519 3.798789 −3.911372  H 1.196131 6.170875 −3.189474  C −1.684052 2.685856 1.410337  C −3.022760 2.949811 1.737008  C −0.673074 3.058419 2.313253  C −3.344505 3.581604 2.944846  H −3.820285 2.666778 1.048578  C −0.996335 3.697772 3.512124  H 0.371961 2.852366 2.075632  C −2.333759 3.958822 3.833384  H −4.391193 3.781126 3.186386  H −0.200071 3.983303 4.203249  H −2.585970 4.452438 4.774752  H 3.725204 1.823050 −1.289692  H 2.104324 0.318087 −1.335647 |
| **Ru−I-II** | |
| 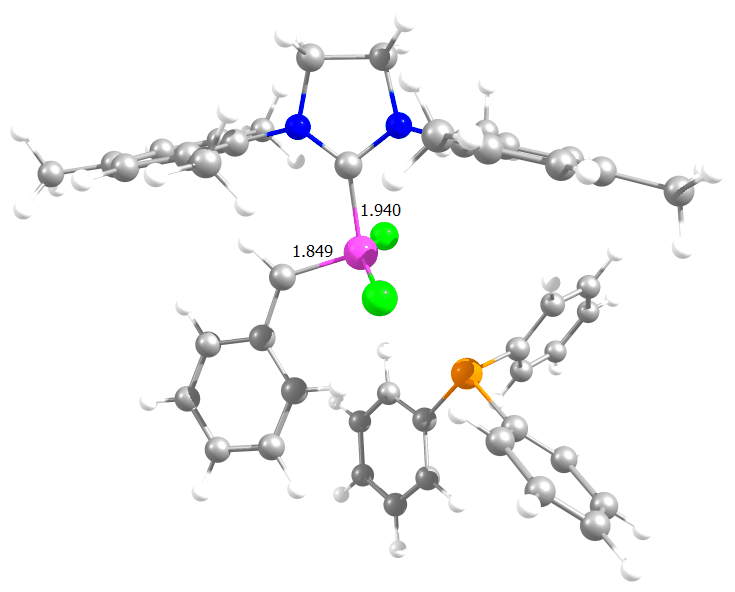  Zero-point correction= 0.779424 (Hartree/Particle)  Thermal correction to Energy= 0.834154  Thermal correction to Enthalpy= 0.835098  Thermal correction to Gibbs Free Energy= 0.681000  Sum of electronic and Zero-point Energies= −3247.486146  Sum of electronic and thermal Energies= −3247.431415  Sum of electronic and thermal Enthalpies= −3247.430471  Sum of electronic and thermal Free Energies= −3247.584570 | Ru −0.927617 −0.167812 −0.091135  Cl −0.380406 −0.486282 −2.347989  Cl −0.421952 −0.039715 2.198125  P 2.853973 0.722765 0.156837  N −2.004933 −2.842619 0.200587  N −3.664612 −1.402892 0.412010  C −2.317963 −1.499598 0.147348  C −3.123485 −3.671982 0.690847  H −2.953997 −3.958136 1.743286  H −3.214735 −4.588217 0.090257  C −4.316159 −2.727980 0.533446  H −4.908554 −2.943753 −0.373411  H −4.994452 −2.741084 1.398304  C −0.745908 −3.473037 −0.101280  C −0.521975 −3.916216 −1.427116  C −1.554894 −3.721661 −2.507841  H −1.240713 −4.223580 −3.433453  H −1.696945 −2.653953 −2.728618  H −2.531930 −4.138126 −2.212524  C 0.675132 −4.582776 −1.713147  H 0.858937 −4.912325 −2.739926  C 1.630095 −4.853823 −0.722846  C 2.933463 −5.533578 −1.067963  H 3.337386 −6.091080 −0.210221  H 3.693559 −4.792658 −1.366748  H 2.812476 −6.233477 −1.907971  C 1.339940 −4.474000 0.592912  H 2.046273 −4.723540 1.390630  C 0.155055 −3.803001 0.936016  C −0.148646 −3.527877 2.387370  H 0.778330 −3.491379 2.976343  H −0.772973 −4.334332 2.810379  H −0.669390 −2.572163 2.528934  C −4.540207 −0.267117 0.348516  C −4.933163 0.354400 1.552596  C −4.345833 −0.067509 2.876586  H −4.743266 0.553247 3.691423  H −3.247756 0.020192 2.873229  H −4.580877 −1.118424 3.112101  C −5.858747 1.406601 1.482429  H −6.161063 1.902304 2.409794  C −6.400845 1.839069 0.264132  C −7.425088 2.948758 0.218613  H −8.449195 2.539103 0.214697  H −7.315970 3.560239 −0.689220  H −7.340536 3.611568 1.091902  C −5.987163 1.196121 −0.912280  H −6.389010 1.527824 −1.874374  C −5.065754 0.140463 −0.897848  C −4.610589 −0.497181 −2.187171  H −4.800467 −1.582243 −2.200835  H −3.528280 −0.358638 −2.338641  H −5.136728 −0.054887 −3.044137  C −1.948939 1.364938 −0.251946  C −2.321771 3.793187 −0.406478  C −1.481481 2.678943 −0.667773  C −1.942357 5.081033 −0.778481  C −0.253549 2.911410 −1.334007  C −0.720413 5.288784 −1.432889  H −2.600359 5.926662 −0.564881  C 0.116635 4.200488 −1.712268  H 0.389813 2.063841 −1.575218  H −0.423588 6.297085 −1.730474  H 1.066721 4.360539 −2.225787  C 3.842279 −0.050287 1.538377  C 4.661358 0.670755 2.423044  C 3.736263 −1.444393 1.695102  C 5.370323 0.009622 3.433486 |

**Table S1.** *Cont.*

| **Ru−I-II** | |
| --- | --- |
|  | H 4.743644 1.755047 2.324607  C 4.454556 −2.104139 2.696153  H 3.082044 −2.014092 1.029604  C 5.273030 −1.378802 3.570557  H 6.002502 0.584555 4.114753  H 4.367333 −3.188226 2.802453  H 5.827112 −1.893412 4.358985  C 3.033636 2.537530 0.539891  C 3.884229 3.414597 −0.152828  C 2.205781 3.052233 1.557648  C 3.920820 4.775509 0.177830  H 4.522746 3.033731 −0.952368  C 2.257131 4.406875 1.895874  H 1.514980 2.385170 2.081567  C 3.114002 5.273675 1.205815  H 4.588521 5.446209 −0.368916  H 1.613910 4.789913 2.691563  H 3.147082 6.334519 1.464853  C 4.008859 0.485490 −1.289491  C 5.409011 0.416903 −1.176139  C 3.419690 0.346295 −2.559091  C 6.203638 0.232721 −2.312601  H 5.878852 0.499418 −0.193407  C 4.217257 0.168925 −3.695875  H 2.329708 0.350302 −2.651381  C 5.609778 0.113016 −3.575351  H 7.290550 0.180101 −2.211262  H 3.745770 0.060020 −4.675468  H 6.232154 −0.033093 −4.461413  H −3.278101 3.624312 0.095557  H −3.001250 1.357413 0.061069 |

**Table S1.** *Cont.*

| **Ru−II** | |
| --- | --- |
| 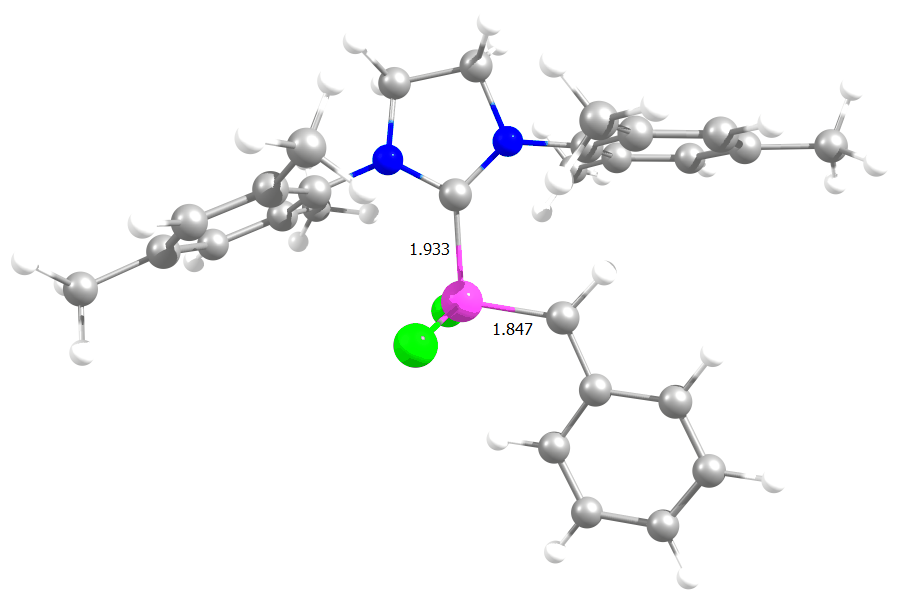  Zero-point correction= 0.514198 (Hartree/Particle)  Thermal correction to Energy= 0.551105  Thermal correction to Enthalpy= 0.552049  Thermal correction to Gibbs Free Energy= 0.440598  Sum of electronic and Zero-point Energies= −2211.214928  Sum of electronic and thermal Energies= −2211.178021  Sum of electronic and thermal Enthalpies= −2211.177077  Sum of electronic and thermal Free Energies= −2211.288528 | Ru −0.228253 0.906791 0.085788  Cl −0.376060 1.238734 2.387216  Cl −0.998424 1.565050 −2.004403  N −1.804914 −1.517052 0.204079  N 0.334620 −2.068650 0.217082  C −0.528071 −1.002684 0.126511  C −1.824349 −2.957524 0.522647  H −2.544065 −3.483359 −0.121523  H −2.120261 −3.113781 1.574449  C −0.371512 −3.370035 0.260440  H 0.046231 −4.002030 1.057194  H −0.251002 −3.902190 −0.699508  C −3.044297 −0.801887 0.044972  C −3.761946 −0.354941 1.176589  C −3.307058 −0.638474 2.586652  H −3.715810 0.107377 3.282479  H −2.214668 −0.619331 2.685129  H −3.671989 −1.627076 2.916677  C −4.976476 0.316977 0.965334  H −5.524604 0.689156 1.836121  C −5.509897 0.508694 −0.313890  C −6.794815 1.275731 −0.515781  H −7.392253 0.852165 −1.336934  H −6.585089 2.327276 −0.773769  H −7.411844 1.276834 0.394442  C −4.813835 −0.026617 −1.407827  H −5.234156 0.074461 −2.412761  C −3.593101 −0.693203 −1.255449  C −2.902973 −1.292531 −2.454802  H −3.557814 −1.247454 −3.336037  H −2.633840 −2.347496 −2.285511  H −1.978990 −0.742680 −2.688944  C 1.760168 −2.100420 0.060634  C 2.314810 −2.206459 −1.233564  C 1.448481 −2.141682 −2.468113  H 2.056977 −2.265424 −3.374390  H 0.924064 −1.175126 −2.536915  H 0.675582 −2.926261 −2.474213  C 3.708018 −2.316614 −1.349374  H 4.147235 −2.390828 −2.348822  C 4.548232 −2.330259 −0.226419  C 6.043012 −2.488395 −0.377585  H 6.334418 −3.550539 −0.318043  H 6.586188 −1.955965 0.416815  H 6.392677 −2.107509 −1.348137  C 3.961549 −2.218736 1.042317  H 4.600844 −2.214022 1.930163  C 2.574106 −2.107949 1.212811  C 1.975890 −1.961144 2.589310  H 1.264680 −2.772293 2.814721  H 1.420530 −1.014533 2.681661  H 2.761125 −1.980463 3.357479  C 1.596970 1.023110 −0.171581  C 3.676251 2.297365 −0.572299  C 2.331833 2.278220 −0.123036  C 4.417970 3.477974 −0.569266  C 1.769184 3.491643 0.346336  C 3.839686 4.667831 −0.109074  H 5.451598 3.472599 −0.922907  C 2.514786 4.667860 0.350407  H 0.746377 3.500635 0.732099  H 4.420532 5.592825 −0.102737  H 2.065261 5.592555 0.718728  H 4.125302 1.366796 −0.928985  H 2.202499 0.147370 −0.440042 |

**Table S1.** *Cont.*

| **Ru−II-III** | |
| --- | --- |
| 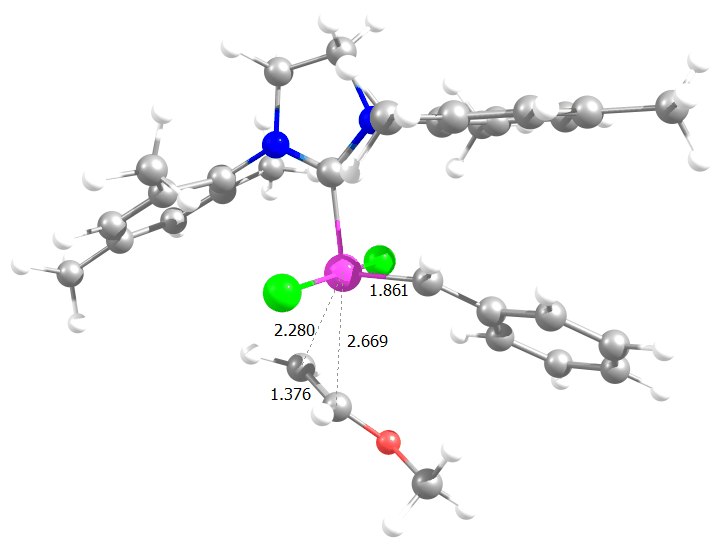  Zero-point correction= 0.597129 (Hartree/Particle)  Thermal correction to Energy= 0.640231  Thermal correction to Enthalpy= 0.641175  Thermal correction to Gibbs Free Energy= 0.515475  Sum of electronic and Zero-point Energies= −2404.311286  Sum of electronic and thermal Energies= −2404.268184  Sum of electronic and thermal Enthalpies= −2404.267240  Sum of electronic and thermal Free Energies= −2404.392940 | Ru −0.234413 0.413977 0.049851  Cl −0.490477 0.637197 2.369763  Cl −0.788716 0.785889 −2.211616  N −1.452571 −2.213597 0.013162  N 0.738527 −2.473379 0.029693  C −0.255769 −1.526465 0.008996  C −1.279729 −3.665602 0.213350  H −1.924680 −4.230423 −0.475086  H −1.546812 −3.944002 1.247747  C 0.213352 −3.854632 −0.070706  H 0.714021 −4.506064 0.659905  H 0.398433 −4.260010 −1.080528  C −2.776539 −1.646791 0.039053  C −3.420233 −1.391245 1.271047  C −2.814134 −1.768402 2.600159  H −3.108775 −1.053277 3.380299  H −1.719258 −1.785396 2.573297  H −3.178014 −2.764093 2.910106  C −4.712004 −0.842065 1.241506  H −5.203384 −0.614849 2.192399  C −5.390614 −0.597017 0.042741  C −6.759031 0.040945 0.037802  H −7.389957 −0.364868 −0.767055  H −6.681795 1.128936 −0.124732  H −7.279075 −0.112378 0.994471  C −4.762870 −0.961134 −1.158031  H −5.296961 −0.830299 −2.103812  C −3.472277 −1.502432 −1.186967  C −2.869200 −1.950339 −2.493774  H −3.608318 −1.871165 −3.303172  H −2.533115 −2.998788 −2.444028  H −1.998239 −1.332284 −2.758949  C 2.160066 −2.302037 −0.060458  C 2.778380 −2.278913 −1.329357  C 1.964007 −2.289802 −2.600438  H 2.611217 −2.134302 −3.474403  H 1.197900 −1.498158 −2.593376  H 1.439529 −3.248461 −2.745612  C 4.176941 −2.183669 −1.379933  H 4.665725 −2.151560 −2.358495  C 4.959987 −2.116426 −0.218289  C 6.466977 −2.048987 −0.301822  H 6.797735 −1.590095 −1.244903  H 6.909468 −3.058284 −0.255361  H 6.891099 −1.468328 0.530422  C 4.309083 −2.137611 1.023551  H 4.901669 −2.071924 1.940851  C 2.914471 −2.235546 1.129700  C 2.240398 −2.231749 2.478762  H 1.616102 −3.128281 2.623705  H 1.577093 −1.358914 2.591511  H 2.986446 −2.206708 3.284980  C 1.578064 0.749944 −0.083438  C 3.534054 2.231752 −0.314851  C 2.213218 2.036650 0.166185  C 4.197789 3.441639 −0.120870  C 1.594360 3.099947 0.869440  C 3.565850 4.483300 0.572200  H 5.212874 3.574305 −0.502562  C 2.267243 4.304524 1.068713  H 0.593553 2.959428 1.281174  H 4.088304 5.429298 0.732843  H 1.776162 5.109456 1.619646  C −1.847670 3.772652 −0.526540  C −2.360454 2.942309 0.394760  H −2.853637 2.028444 0.067023  H −2.345476 3.172834 1.459756  H −1.845484 3.520025 −1.592962  O −1.290061 4.977319 −0.199077  C −0.640727 5.649174 −1.290170  H −0.312542 6.621206 −0.902611  H 0.234838 5.080070 −1.642878  H −1.340046 5.809676 −2.128781  H 4.026097 1.413215 −0.846641  H 2.268617 −0.013014 −0.467435 |

**Table S1.** *Cont.*

| **Ru−IIt** | |
| --- | --- |
| 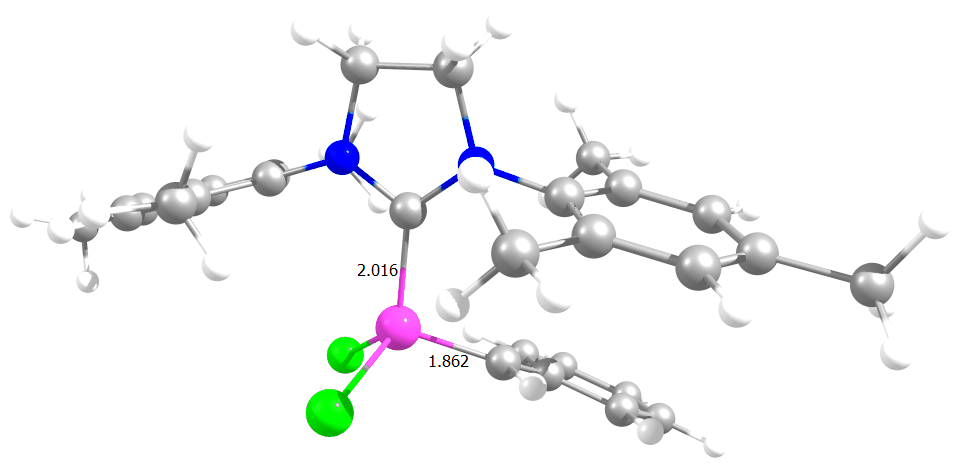  Zero-point correction= 0.512787 (Hartree/Particle)  Thermal correction to Energy= 0.550292  Thermal correction to Enthalpy= 0.551236  Thermal correction to Gibbs Free Energy= 0.437705  Sum of electronic and Zero-point Energies= −2211.185941  Sum of electronic and thermal Energies= −2211.148436  Sum of electronic and thermal Enthalpies= −2211.147492  Sum of electronic and thermal Free Energies= −2211.261023 | Ru −0.359707 0.603387 −0.859840  Cl −1.531775 2.608520 −0.602707  Cl −0.816863 −0.346755 −2.981163  N −1.674085 −1.384131 0.892557  N 0.493675 −1.783456 0.899451  C −0.473577 −0.952443 0.416524  C −1.553440 −2.538076 1.812158  H −2.224217 −3.352231 1.500296  H −1.834116 −2.236886 2.835256  C −0.065561 −2.908902 1.690809  H 0.441176 −2.979406 2.664719  H 0.089309 −3.859498 1.154661  C −2.943126 −0.733776 0.693810  C −3.326657 0.317139 1.554657  C −2.400601 0.840372 2.624103  H −2.926269 1.553122 3.274096  H −1.545758 1.365495 2.169169  H −1.996392 0.036597 3.259069  C −4.588026 0.895164 1.369730  H −4.887008 1.720034 2.023000  C −5.468030 0.454190 0.371549  C −6.801834 1.130379 0.163247  H −7.538411 0.443021 −0.277490  H −6.698740 1.987538 −0.522871  H −7.211286 1.515423 1.108748  C −5.064569 −0.609079 −0.446484  H −5.741124 −0.974306 −1.224686  C −3.812391 −1.223350 −0.305091  C −3.412874 −2.354981 −1.218361  H −4.265845 −2.670380 −1.834885  H −3.057562 −3.235360 −0.659843  H −2.599739 −2.043302 −1.894602  C 1.897023 −1.781050 0.595884  C 2.369871 −2.482086 −0.534254  C 1.424932 −3.145557 −1.505342  H 1.985844 −3.659643 −2.297682  H 0.754022 −2.412225 −1.982668  H 0.784890 −3.894103 −1.011203  C 3.754899 −2.521424 −0.752538  H 4.130680 −3.055084 −1.630763  C 4.664647 −1.903572 0.117310  C 6.153265 −1.999635 −0.121053  H 6.579463 −2.873913 0.399110  H 6.678984 −1.109094 0.253236  H 6.383684 −2.111457 −1.190494  C 4.157099 −1.215910 1.228591  H 4.848916 −0.713576 1.910930  C 2.782472 −1.145124 1.490911  C 2.266446 −0.373981 2.680049  H 1.623764 −0.991868 3.326947  H 1.663218 0.488043 2.355415  H 3.098437 0.002842 3.290254  C 1.461772 0.986569 −0.895264  C 3.638521 2.119694 −0.738962  C 2.263785 2.080399 −0.383319  C 4.474374 3.131270 −0.269013  C 1.766164 3.103319 0.463176  C 3.962031 4.134435 0.563546  H 5.527710 3.143712 −0.558521  C 2.605077 4.116224 0.920017  H 0.706707 3.102490 0.724504  H 4.614133 4.931610 0.927358  H 2.200373 4.902786 1.561050  H 4.035835 1.341276 −1.394866  H 2.005977 0.291170 −1.560884 |

**Table S1.** *Cont.*

| **Ru−III** | |
| --- | --- |
| 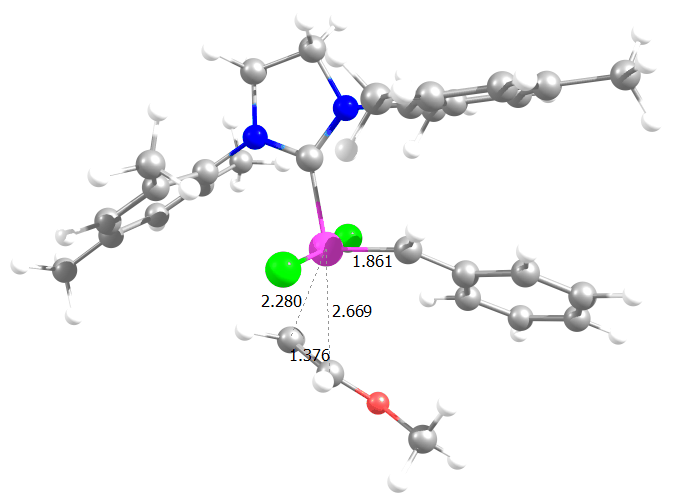  Zero-point correction= 0.598571 (Hartree/Particle)  Thermal correction to Energy= 0.640444  Thermal correction to Enthalpy= 0.641388  Thermal correction to Gibbs Free Energy= 0.521763  Sum of electronic and Zero-point Energies= −2404.320309  Sum of electronic and thermal Energies= −2404.278435  Sum of electronic and thermal Enthalpies= −2404.277491  Sum of electronic and thermal Free Energies= −2404.397116 | Ru −0.420268 0.738365 −0.196777  Cl −0.429014 1.106341 2.181479  Cl −0.729693 0.491439 −2.608135  N −1.942578 −1.741863 0.300783  N 0.187891 −2.279697 0.191469  C −0.686057 −1.239798 0.098878  C −1.944190 −3.176608 0.661872  H −2.662245 −3.727177 0.036616  H −2.235926 −3.302529 1.717441  C −0.486946 −3.585051 0.402883  H −0.031337 −4.112929 1.253141  H −0.377397 −4.217362 −0.493612  C −3.184255 −1.008789 0.262224  C −3.754167 −0.517763 1.460768  C −3.153637 −0.798090 2.815252  H −3.539108 −0.089655 3.561424  H −2.059729 −0.710390 2.809952  H −3.429331 −1.812204 3.154565  C −4.964119 0.187685 1.376983  H −5.395454 0.591387 2.297832  C −5.641262 0.367535 0.164512  C −6.918608 1.170646 0.098726  H −7.587870 0.799188 −0.691303  H −6.705658 2.229530 −0.124701  H −7.462544 1.141087 1.054093  C −5.103232 −0.226700 −0.985305  H −5.647522 −0.154710 −1.931614  C −3.895253 −0.937395 −0.961274  C −3.409890 −1.636432 −2.204570  H −4.175225 −1.589243 −2.991842  H −3.191571 −2.698708 −2.009194  H −2.487649 −1.171801 −2.588054  C 1.617132 −2.281521 0.046590  C 2.181771 −2.554938 −1.217314  C 1.324907 −2.699893 −2.450422  H 1.950805 −2.881660 −3.335085  H 0.720446 −1.795274 −2.633173  H 0.620152 −3.543086 −2.365222  C 3.578428 −2.665044 −1.306459  H 4.026133 −2.874847 −2.282645  C 4.408303 −2.523957 −0.186594  C 5.909628 −2.644323 −0.306480  H 6.403738 −1.684252 −0.085846  H 6.208247 −2.952055 −1.318576  H 6.309830 −3.383640 0.405036  C 3.809848 −2.261477 1.054490  H 4.440330 −2.147893 1.941377  C 2.421513 −2.144097 1.200032  C 1.811856 −1.871166 2.551174  H 1.119251 −2.674084 2.853322  H 1.232833 −0.933288 2.551033  H 2.593737 −1.798121 3.319624  C 1.417764 0.727181 −0.487327  C 3.809894 1.267367 −0.511166  C 2.539427 1.452460 0.099691  C 4.942257 1.929175 −0.041057  C 2.456752 2.337251 1.202991  C 4.838661 2.796459 1.055681  H 5.907680 1.772871 −0.528164  C 3.596219 2.991293 1.673170  H 1.492230 2.484030 1.688318  H 5.724310 3.317466 1.427459  H 3.513828 3.664784 2.529532  C −1.407677 2.793059 −0.258725  C −0.368923 3.275941 −1.021354  H −2.265378 2.378445 −0.800304  H −1.566240 3.166219 0.753820  H −0.269021 3.014605 −2.080881  O 0.521552 4.155070 −0.519319  C 1.580187 4.532442 −1.427401  H 1.160532 5.020879 −2.321527  H 2.211586 5.240059 −0.879421  H 2.174141 3.653925 −1.718868  H 3.887787 0.586711 −1.362741  H 1.733836 0.121033 −1.354478 |

**Table S1.** *Cont.*

| **Ru−III-IV** | |
| --- | --- |
| 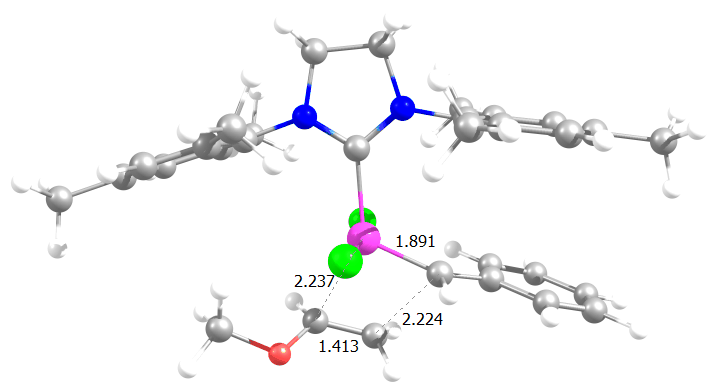  Zero-point correction= 0.599147 (Hartree/Particle)  Thermal correction to Energy= 0.640593  Thermal correction to Enthalpy= 0.641537  Thermal correction to Gibbs Free Energy= 0.523596  Sum of electronic and Zero-point Energies= −2404.309487  Sum of electronic and thermal Energies= −2404.268041  Sum of electronic and thermal Enthalpies= −2404.267097  Sum of electronic and thermal Free Energies= −2404.385038 | Ru 0.243591 −0.882958 −0.089555  Cl 0.095499 −1.111710 2.327454  Cl 0.790048 −0.716681 −2.451180  N 1.719808 1.748432 0.353764  N −0.444093 2.092027 0.165909  C 0.521473 1.138900 0.133900  C 1.576443 3.192232 0.670198  H 2.275662 3.786139 0.064294  H 1.802467 3.367483 1.734775  C 0.105936 3.461644 0.331256  H −0.429187 3.992399 1.131417  H −0.018889 4.031078 −0.604916  C 3.038648 1.165861 0.322587  C 3.647440 0.731654 1.522822  C 2.963322 0.853339 2.860718  H 3.533612 0.318248 3.632774  H 1.942906 0.443208 2.844050  H 2.900961 1.909305 3.175041  C 4.955936 0.227356 1.455062  H 5.427297 −0.122523 2.378451  C 5.681162 0.188859 0.257580  C 7.078274 −0.383592 0.211318  H 7.694746 0.121053 −0.547129  H 7.057852 −1.455886 −0.046266  H 7.584092 −0.290863 1.183461  C 5.073896 0.700405 −0.897690  H 5.638463 0.724738 −1.834655  C 3.766880 1.208066 −0.890626  C 3.182936 1.803119 −2.145756  H 3.936433 1.828036 −2.945163  H 2.833688 2.835529 −1.981896  H 2.321350 1.211507 −2.495745  C −1.858274 1.946962 −0.060251  C −2.361417 2.026565 −1.376682  C −1.449161 2.153097 −2.570171  H −2.036760 2.267603 −3.491755  H −0.798227 1.268936 −2.680470  H −0.786255 3.029948 −2.488497  C −3.752672 1.982780 −1.552359  H −4.152349 2.039878 −2.569385  C −4.637908 1.884212 −0.471026  C −6.129633 1.807147 −0.692727  H −6.683242 2.332339 0.099927  H −6.468692 0.757826 −0.688701  H −6.416275 2.244464 −1.660177  C −4.100582 1.837333 0.822530  H −4.774990 1.771670 1.681447  C −2.719052 1.875343 1.055618  C −2.180929 1.843981 2.464110  H −1.668916 2.785812 2.724732  H −1.452999 1.028692 2.601427  H −2.999165 1.705688 3.184428  C −1.458446 −1.453483 −0.682811  C −3.846250 −1.928515 −0.966044  C −2.767140 −1.716673 −0.071985  C −5.127705 −2.217899 −0.496066  C −3.024160 −1.805472 1.314334  C −5.359183 −2.314167 0.881149  H −5.943277 −2.381426 −1.204282  C −4.303103 −2.109845 1.779650  H −2.201155 −1.643248 2.013049  H −6.358411 −2.552123 1.253718  H −4.480304 −2.187201 2.854919  C −0.158777 −3.177204 −0.148211  C 1.224757 −2.893725 −0.100308  H −0.672225 −3.395024 0.787399  H −0.523181 −3.665929 −1.054519  H 1.724420 −2.789155 0.874905  O 2.008794 −3.308598 −1.131398  C 3.341952 −2.768324 −1.150764  H 3.789023 −2.788064 −0.141644  H 3.921762 −3.408477 −1.826508  H 3.321425 −1.737886 −1.533734  H −3.660485 −1.868595 −2.041558  H −1.501796 −1.556568 −1.782095 |

**Table S1.** *Cont.*

| **Ru−IV** | |
| --- | --- |
| 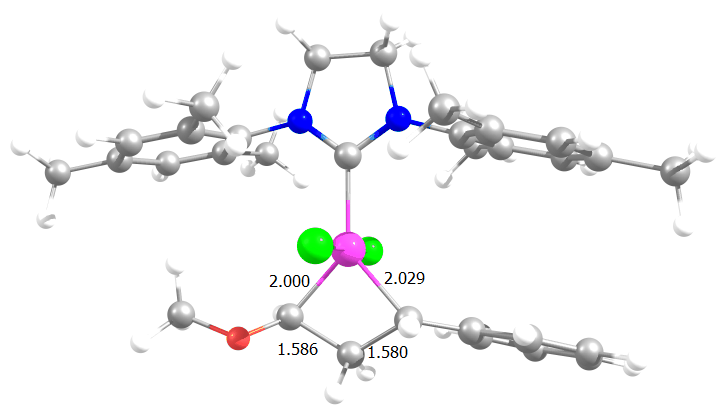  Zero-point correction= 0.601099 (Hartree/Particle)  Thermal correction to Energy= 0.642794  Thermal correction to Enthalpy= 0.643739  Thermal correction to Gibbs Free Energy= 0.524387  Sum of electronic and Zero-point Energies= −2404.319871  Sum of electronic and thermal Energies= −2404.278176  Sum of electronic and thermal Enthalpies= −2404.277231  Sum of electronic and thermal Free Energies= −2404.396583 | Ru 0.173013 −0.824770 −0.204746  Cl −0.024636 −1.283528 2.177176  Cl 0.515163 −0.376059 −2.563308  N 1.780752 1.667380 0.460107  N −0.376341 2.096785 0.306438  C 0.563628 1.127270 0.195856  C 1.686624 3.091628 0.873340  H 2.381103 3.701728 0.277969  H 1.960450 3.191901 1.935789  C 0.212249 3.425981 0.609848  H −0.289513 3.868811 1.481911  H 0.074513 4.102179 −0.249037  C 3.088227 1.063330 0.378091  C 3.685013 0.531621 1.543666  C 2.968915 0.510342 2.869429  H 3.557924 −0.042329 3.614451  H 1.976813 0.038356 2.793659  H 2.823261 1.531582 3.260604  C 4.999511 0.048047 1.450146  H 5.464578 −0.372827 2.346624  C 5.737507 0.115887 0.261632  C 7.143833 −0.430684 0.187012  H 7.760064 0.139910 −0.523279  H 7.142824 −1.480172 −0.152341  H 7.637799 −0.405267 1.169228  C 5.131220 0.699503 −0.859712  H 5.699643 0.791572 −1.790028  C 3.817880 1.191296 −0.826839  C 3.224340 1.840564 −2.049403  H 3.979423 1.929800 −2.842554  H 2.843447 2.851855 −1.832742  H 2.377120 1.248888 −2.435007  C −1.802234 2.004538 0.097672  C −2.331122 2.282751 −1.182190  C −1.446865 2.585463 −2.364347  H −2.055434 2.760915 −3.262329  H −0.753786 1.753663 −2.571790  H −0.838521 3.490080 −2.197850  C −3.726204 2.288663 −1.329779  H −4.144862 2.497148 −2.318928  C −4.591113 2.058436 −0.252935  C −6.088346 2.046640 −0.446996  H −6.609415 2.505975 0.406425  H −6.460207 1.012461 −0.537131  H −6.380820 2.585684 −1.359719  C −4.029462 1.829003 1.010005  H −4.687354 1.669217 1.869093  C −2.643618 1.809923 1.216462  C −2.089590 1.604564 2.602628  H −1.453114 2.448301 2.917043  H −1.472748 0.693243 2.658297  H −2.907070 1.516331 3.331458  C −1.401857 −1.929529 −0.849576  C −3.857854 −1.763911 −1.030657  C −2.738889 −2.062760 −0.222283  C −5.159678 −1.921610 −0.550629  C −2.972098 −2.513816 1.093491  C −5.373596 −2.379605 0.753836  H −6.008502 −1.698258 −1.201317  C −4.273459 −2.671130 1.571430  H −2.123529 −2.707471 1.750926  H −6.390114 −2.509345 1.132522  H −4.431883 −3.022428 2.593788  C −0.296895 −3.019150 −0.552036  C 1.191568 −2.543994 −0.281126  H −0.609377 −3.616137 0.311688  H −0.203975 −3.602295 −1.478192  H 1.562899 −2.825264 0.719384  O 2.035456 −2.926788 −1.296143  C 3.429289 −2.767827 −0.993682  H 3.674493 −3.221711 −0.016784  H 3.976937 −3.285801 −1.790161  H 3.704170 −1.702170 −0.985140  H −3.692963 −1.415756 −2.053544  H −1.491899 −1.762968 −1.930725 |

**Table S1.** *Cont.*

| **Ru−IV−V** | |
| --- | --- |
| 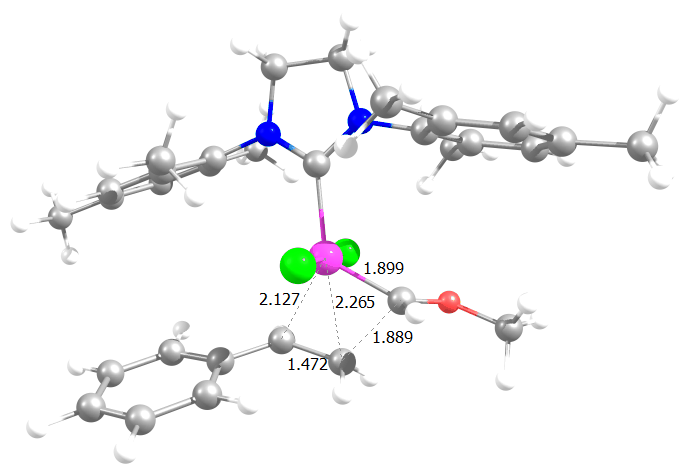  Zero-point correction= 0.599463 (Hartree/Particle)  Thermal correction to Energy= 0.641034  Thermal correction to Enthalpy= 0.641978  Thermal correction to Gibbs Free Energy= 0.522500  Sum of electronic and Zero-point Energies= −2404.319170  Sum of electronic and thermal Energies= −2404.277599  Sum of electronic and thermal Enthalpies= −2404.276655  Sum of electronic and thermal Free Energies= −2404.396133 | Ru −0.137503 0.695245 −0.433672  Cl −0.174001 1.545979 1.841803  Cl −0.056633 −0.136937 −2.704879  N −1.680610 −1.732009 0.586692  N 0.463825 −2.185265 0.383963  C −0.475025 −1.217185 0.234124  C −1.605422 −3.181887 0.901314  H −2.093828 −3.763321 0.101880  H −2.121413 −3.394481 1.848196  C −0.093567 −3.430770 0.969918  H 0.271649 −3.554003 2.003166  H 0.226295 −4.304147 0.384596  C −2.971397 −1.091328 0.571121  C −3.493519 −0.598300 1.789049  C −2.699175 −0.648084 3.068982  H −3.290462 −0.247705 3.904214  H −1.775170 −0.054153 2.977907  H −2.408414 −1.678674 3.330869  C −4.782876 −0.048995 1.781707  H −5.187912 0.345443 2.718568  C −5.567181 −0.007252 0.619773  C −6.971854 0.549763 0.655629  H −7.672073 −0.172822 1.107056  H −7.340815 0.777561 −0.354750  H −7.026056 1.470117 1.256888  C −5.028315 −0.534035 −0.560474  H −5.630853 −0.529670 −1.473841  C −3.741271 −1.093901 −0.612063  C −3.221766 −1.673548 −1.901858  H −4.008718 −1.668479 −2.668828  H −2.885439 −2.715109 −1.775744  H −2.358908 −1.104186 −2.284779  C 1.890254 −2.078665 0.195761  C 2.458356 −2.603083 −0.989326  C 1.606078 −3.196573 −2.080957  H 2.234786 −3.545783 −2.911792  H 0.894558 −2.450381 −2.469697  H 1.022410 −4.060104 −1.720886  C 3.851581 −2.563054 −1.126344  H 4.296891 −2.957515 −2.044821  C 4.684572 −2.050286 −0.120751  C 6.183825 −2.017630 −0.305226  H 6.693002 −1.684000 0.609893  H 6.470000 −1.331299 −1.118851  H 6.577615 −3.011582 −0.570194  C 4.089469 −1.581141 1.055411  H 4.722280 −1.197936 1.860898  C 2.698719 −1.596314 1.248130  C 2.115716 −1.123502 2.555222  H 1.450121 −1.879345 3.002348  H 1.517601 −0.205856 2.430951  H 2.916892 −0.915004 3.277533  C 1.346105 2.061974 −1.107756  C 3.748322 1.871821 −0.564375  C 2.489905 2.424470 −0.243003  C 4.894042 2.218017 0.155161  C 2.419221 3.336799 0.830348  C 4.807546 3.129366 1.212737  H 5.857908 1.781290 −0.115714  C 3.564363 3.684392 1.546052  H 1.456713 3.757226 1.121192  H 5.702167 3.408708 1.774058  H 3.487572 4.393152 2.373813  C 0.086896 2.820923 −1.183832  C −1.532317 1.938030 −0.775039  H 0.000931 3.648367 −0.473511  H −0.213153 3.047323 −2.211198  H −1.925277 2.515937 0.082292  O −2.366682 1.912474 −1.839109  C −3.506041 2.801621 −1.751665  H −3.182563 3.853819 −1.804253  H −4.136842 2.566616 −2.616442  H −4.068385 2.623482 −0.822195  H 3.817706 1.163653 −1.394139  H 1.659597 1.639637 −2.069155 |

**Table S1.** *Cont.*

| **Ru−V** | |
| --- | --- |
| 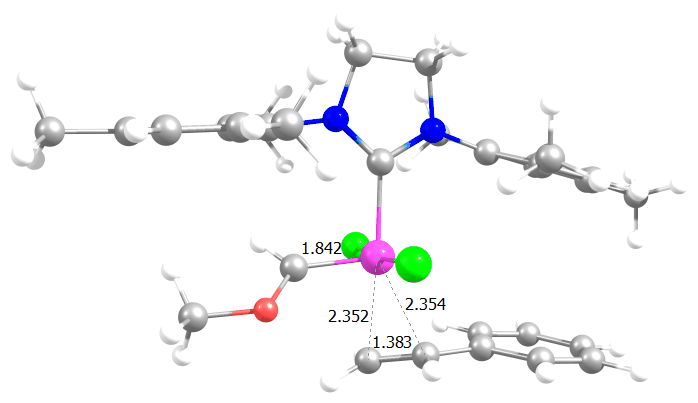  Zero-point correction= 0.599611 (Hartree/Particle)  Thermal correction to Energy= 0.642151  Thermal correction to Enthalpy= 0.643095  Thermal correction to Gibbs Free Energy= 0.520382  Sum of electronic and Zero-point Energies= −2404.328653  Sum of electronic and thermal Energies= −2404.286113  Sum of electronic and thermal Enthalpies= −2404.285169  Sum of electronic and thermal Free Energies= −2404.407883 | Ru −0.091074 −0.961943 −0.098492  Cl 0.128480 −0.595736 −2.502292  Cl −0.176607 −1.161074 2.327664  N −1.806081 1.632182 0.278152  N 0.355383 1.987089 0.394263  C −0.587651 1.028850 0.169293  C −1.715306 3.095262 0.516080  H −2.354610 3.381800 1.363330  H −2.060267 3.644954 −0.375909  C −0.223627 3.292980 0.795861  H 0.224637 4.103852 0.204209  H −0.014642 3.482878 1.861091  C −3.124052 1.091915 0.087329  C −3.697409 1.110141 −1.205350  C −2.906774 1.542682 −2.414152  H −3.533614 1.510062 −3.315976  H −2.026902 0.897004 −2.578683  H −2.528551 2.572006 −2.307241  C −5.027742 0.686645 −1.345221  H −5.475104 0.689444 −2.343870  C −5.799427 0.278736 −0.246957  C −7.246152 −0.120672 −0.422814  H −7.903350 0.764767 −0.399499  H −7.579130 −0.795425 0.379150  H −7.412412 −0.622782 −1.387387  C −5.201903 0.287125 1.020688  H −5.786993 −0.023214 1.891678  C −3.874253 0.699273 1.217735  C −3.278502 0.719091 2.601379  H −4.000208 0.341073 3.338698  H −2.999181 1.742018 2.904433  H −2.362605 0.107574 2.655679  C 1.789210 1.910039 0.244826  C 2.607522 1.720810 1.382077  C 2.034409 1.536040 2.763837  H 2.818146 1.212964 3.463277  H 1.227212 0.788388 2.780573  H 1.625671 2.485621 3.151271  C 3.998667 1.768671 1.208515  H 4.637464 1.607577 2.081760  C 4.588414 2.040727 −0.031369  C 6.089179 2.065284 −0.189230  H 6.398589 2.740896 −1.000259  H 6.468907 1.059304 −0.434178  H 6.587448 2.386726 0.737300  C 3.746094 2.287731 −1.123003  H 4.184795 2.541230 −2.092696  C 2.350318 2.247444 −1.010115  C 1.490924 2.597457 −2.197146  H 0.780531 3.406640 −1.960575  H 0.904085 1.725703 −2.528336  H 2.115584 2.935691 −3.035622  C 1.347915 −2.793021 0.230601  C 3.628463 −2.364815 1.092826  C 2.737065 −2.349846 −0.001457  C 4.987641 −2.101469 0.914655  C 3.241650 −2.008017 −1.274446  C 5.480863 −1.800586 −0.360200  H 5.665488 −2.140086 1.770565  C 4.599553 −1.738368 −1.447767  H 2.560758 −1.944252 −2.125763  H 6.547447 −1.613702 −0.506271  H 4.975349 −1.484728 −2.441686  C 0.436937 −3.164308 −0.742138  C −1.809099 −1.599380 −0.286887  H 0.669909 −3.117351 −1.806237  H −0.413375 −3.779918 −0.452136  H −2.481935 −1.196337 −1.068400  O −2.347867 −2.602316 0.403566  C −3.675880 −3.055761 0.016507  H −3.578575 −3.869480 −0.716744  H −4.151188 −3.428704 0.930772  H −4.260085 −2.224605 −0.403506  H 3.240831 −2.608330 2.085108  H 1.130843 −3.068342 1.265219 |

**Table S1.** *Cont.*

| **Ru−V−VI** | |
| --- | --- |
| 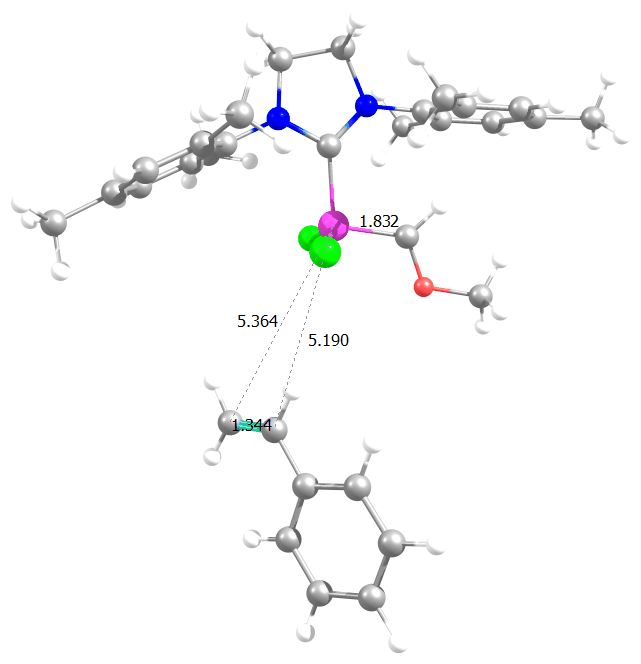  Zero-point correction= 0.596541 (Hartree/Particle)  Thermal correction to Energy= 0.640249  Thermal correction to Enthalpy= 0.641193  Thermal correction to Gibbs Free Energy= 0.508159  Sum of electronic and Zero-point Energies= −2404.323518  Sum of electronic and thermal Energies= −2404.279810  Sum of electronic and thermal Enthalpies= −2404.278865  Sum of electronic and thermal Free Energies= −2404.411899 | Ru 0.190222 0.000682 −0.440583  Cl −1.096392 0.078345 1.520158  Cl 0.415129 0.379954 −2.738769  N 3.141619 0.166049 0.255255  N 2.041759 2.080200 0.312454  C 1.894132 0.724830 0.105014  C 4.154848 1.150864 0.696993  H 5.083157 1.029051 0.120765  H 4.390251 1.006700 1.766505  C 3.454426 2.488307 0.431395  H 3.580521 3.211092 1.250329  H 3.800077 2.957709 −0.505945  C 3.498683 −1.221890 0.276408  C 3.380757 −1.955342 1.477504  C 2.765711 −1.343569 2.712824  H 2.764400 −2.064024 3.542168  H 1.725771 −1.030379 2.526678  H 3.314720 −0.449544 3.047888  C 3.800560 −3.293631 1.479969  H 3.707615 −3.871508 2.404525  C 4.331205 −3.908021 0.335623  C 4.818982 −5.337204 0.380241  H 5.876770 −5.380766 0.689578  H 4.747316 −5.819442 −0.605533  H 4.244428 −5.937816 1.100562  C 4.425284 −3.150964 −0.841001  H 4.826178 −3.616298 −1.746515  C 4.020966 −1.808375 −0.895850  C 4.105582 −1.028399 −2.183917  H 4.541961 −1.644082 −2.982500  H 4.730040 −0.127300 −2.073841  H 3.108803 −0.688715 −2.509151  C 0.992934 3.066267 0.302659  C 0.707479 3.794289 −0.874830  C 1.505691 3.619588 −2.142696  H 0.932682 3.971321 −3.011686  H 1.770951 2.570352 −2.324739  H 2.434102 4.215943 −2.100085  C −0.313735 4.756078 −0.825432  H −0.556112 5.306135 −1.739639  C −1.014317 5.039921 0.352686  C −2.140766 6.045375 0.366417  H −2.171940 6.602733 1.314537  H −3.115225 5.541684 0.252764  H −2.046224 6.769059 −0.456120  C −0.644907 4.360026 1.522478  H −1.148466 4.598990 2.463754  C 0.359629 3.385342 1.528199  C 0.763281 2.722025 2.820427  H 1.852921 2.768158 2.976646  H 0.466431 1.662629 2.825529  H 0.276953 3.216267 3.672895  C −4.993501 −0.133780 −0.227649  C −5.744553 −2.175588 0.968332  C −6.006434 −1.174877 0.009525  C −6.670156 −3.189897 1.229477  C −7.237586 −1.228544 −0.678706  C −7.884334 −3.227417 0.536544  H −6.442476 −3.952785 1.977856  C −8.162019 −2.239563 −0.419137  H −7.473754 −0.468577 −1.426791  H −8.610831 −4.017849 0.737804  H −9.108462 −2.260285 −0.964960  C −5.058588 0.878893 −1.109006  C 0.542008 −1.797025 −0.441330  H −5.914312 1.035924 −1.770417  H −4.235482 1.588698 −1.200109  H 1.451264 −2.314214 −0.091339  O −0.407231 −2.621740 −0.909108  C −0.195491 −4.051965 −0.787640  H −0.680908 −4.411165 0.131071  H −0.663692 −4.514658 −1.664242  H 0.880747 −4.282114 −0.764027  H −4.796366 −2.148518 1.512124  H −4.092561 −0.217807 0.391506 |

**Table S1.** *Cont.*

| **Ru−VI** | |
| --- | --- |
| 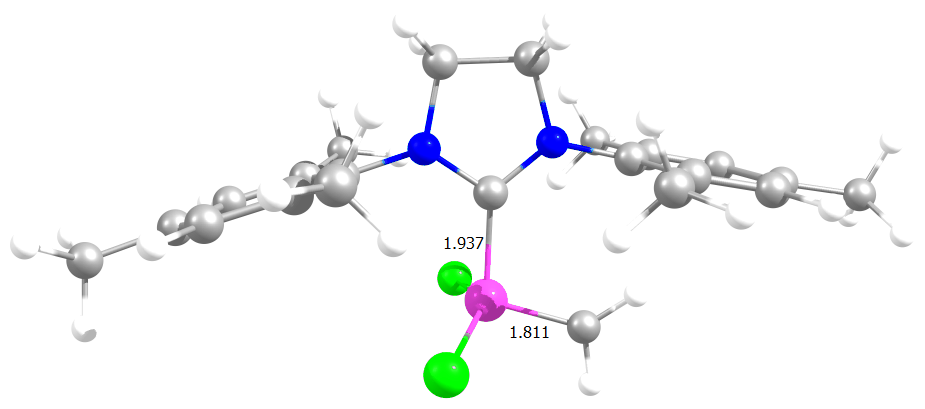  Zero-point correction= 0.435280 (Hartree/Particle)  Thermal correction to Energy= 0.467033  Thermal correction to Enthalpy= 0.467978  Thermal correction to Gibbs Free Energy= 0.370356  Sum of electronic and Zero-point Energies= −1980.161085  Sum of electronic and thermal Energies= −1980.129331  Sum of electronic and thermal Enthalpies= −1980.128387  Sum of electronic and thermal Free Energies= −1980.226009 | Ru −0.097077 0.353440 −1.433386  Cl −1.028641 −1.597178 −2.278175  Cl −0.429092 2.640190 −1.197293  N 1.212061 −0.228614 1.219935  N −0.995970 −0.278076 1.271982  C 0.088414 −0.094496 0.442332  C 0.895780 −0.634970 2.608078  H 1.479396 −0.037504 3.322808  H 1.146297 −1.699217 2.760224  C −0.611086 −0.373536 2.694885  H −1.162304 −1.186158 3.189212  H −0.842229 0.571663 3.216053  C 2.590797 −0.206202 0.823954  C 3.216462 −1.403008 0.413179  C 2.435225 −2.686374 0.266102  H 3.068030 −3.477313 −0.158912  H 1.564265 −2.550309 −0.393587  H 2.056161 −3.051229 1.234693  C 4.580061 −1.358156 0.088635  H 5.070357 −2.276911 −0.246933  C 5.325115 −0.172866 0.171673  C 6.799876 −0.159250 −0.154775  H 7.405081 −0.337511 0.749967  H 7.110970 0.810460 −0.569923  H 7.058485 −0.942828 −0.881564  C 4.669535 0.995120 0.586262  H 5.231248 1.932051 0.646891  C 3.308195 1.003997 0.922906  C 2.627816 2.283698 1.340503  H 3.352316 3.107843 1.394730  H 2.150329 2.188847 2.328891  H 1.836421 2.564783 0.627710  C −2.391676 −0.200922 0.922506  C −3.079678 1.029317 1.007804  C −2.432258 2.290479 1.525310  H −2.866593 3.176957 1.042926  H −1.352868 2.316440 1.339442  H −2.607074 2.388405 2.611293  C −4.442481 1.049167 0.671064  H −4.974639 2.004510 0.707684  C −5.137597 −0.109870 0.310103  C −6.591318 −0.052675 −0.094290  H −7.140852 −0.942279 0.248108  H −6.689203 −0.011269 −1.191979  H −7.088932 0.838613 0.314373 |

**Table S2.** %VBur values (by quadrants) for (**a**) **Rh-Id** and (**b**) **Ru-I**.

| (**a**) | **Quadrant** | **V_f** | **V_b** | **V_t** | **%V_f** | **%VBur** |
| --- | --- | --- | --- | --- | --- | --- |
|  | SE | 30.6 | 14.3 | 44.9 | 68.1 | 31.9 |
|  | NE | 30.6 | 14.3 | 44.9 | 68.1 | 31.9 |
|  | NW | 33.0 | 11.8 | 44.8 | 73.7 | 26.3 |
|  | SW | 30.0 | 14.9 | 44.9 | 66.9 | 33.1 |
| (**b**) | SE | 30.7 | 14.1 | 44.9 | 68.5 | 31.5 |
|  | NE | 31.0 | 13.8 | 44.9 | 69.1 | 30.9 |
|  | NW | 32.5 | 12.3 | 44.8 | 72.6 | 27.4 |
|  | SW | 29.8 | 15.0 | 44.9 | 66.5 | 33.5 |

(**a**)
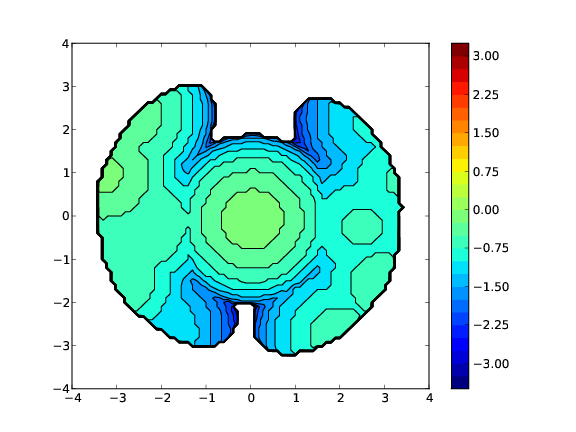


(**b**)
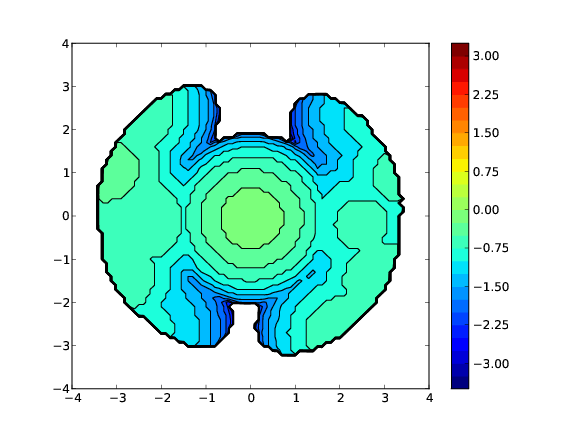


**Figure S1.** Topographic steric maps of the NHC ligands for the studied complexes (**a**) **Rh-Id** and (**b**) **Ru-I**. %VBur is the percent of buried volume. The metal is at the origin and the P atom is on the z axis. The isocontour curves of the steric maps are in Å.
